# Toward the Identification of Natural Antiviral Drug Candidates against Merkel Cell Polyomavirus: Computational Drug Design Approaches

**DOI:** 10.3390/ph15050501

**Published:** 2022-04-20

**Authors:** Amer H. Asseri, Md. Jahidul Alam, Faisal Alzahrani, Ahmed Khames, Mohammad Turhan Pathan, Mohammed A. S. Abourehab, Salman Hosawi, Rubaiat Ahmed, Sifat Ara Sultana, Nazia Fairooz Alam, Nafee-Ul Alam, Rahat Alam, Abdus Samad, Sushil Pokhrel, Jin Kyu Kim, Foysal Ahammad, Bonglee Kim, Shing Cheng Tan

**Affiliations:** 1 Biochemistry Department, Faculty of Science, King Abdul-Aziz University, Jeddah 21589, Saudi Arabia; ahasseri@kau.edu.sa (A.H.A.); faahalzahrani@kau.edu.sa (F.A.); shosawi@kau.edu.sa (S.H.); 2 Centre for Artificial Intelligence in Precision Medicines, King Abdul-Aziz University, Jeddah 21589, Saudi Arabia; 3 Department of Applied Chemistry and Chemical Engineering, Noakhali Science and Technology University, Noakhali 3814, Bangladesh; mdjahidulalam.nstu.bd@gmail.com; 4 King Fahd Medical Research Center, Embryonic Stem Cells Unit, Department of Biochemistry, Faculty of Science, King Abdul-Aziz University, Jeddah 21589, Saudi Arabia; 5 Department of Pharmaceutics and Industrial pharmacy, College of Pharmacy, Taif University, P.O. Box 11099, Taif 21944, Saudi Arabia; dr.akhamies@gmail.com; 6 Department of Biochemistry and Microbiology, North South University, Dhaka 1229, Bangladesh; turhan.pathan@hotmail.com; 7 Department of Pharmaceutics, Faculty of Pharmacy, Umm Al-Qura University, Makkah 21955, Saudi Arabia; maabourehab@uqu.edu.sa; 8 Department of Biochemistry and Molecular Biology, University of Dhaka, Dhaka 1000, Bangladesh; rubaiat1ahmed@gmail.com (R.A.); nazia.fairooz@gmail.com (N.F.A.); 9 Department of Pharmacy, Faculty of Pharmacy, University of Dhaka, Dhaka 1000, Bangladesh; sifatarasultana@gmail.com; 10 Department of Biotechnology, College of Life Science and Medicine, Zhejiang Sci-Tech University, Hangzhou 310018, China; nafee.alam@hotmail.com; 11 Department of Genetic Engineering and Biotechnology, Faculty of Biological Science and Technology, Jashore University of Science and Technology, Jashore 7408, Bangladesh; rahatalam1643@gmail.com (R.A.); kazisamad50@gmail.com (A.S.); 12 Laboratory of Computational Biology, Biological Solution Centre (BioSol Centre), Jashore 7408, Bangladesh; 13 Department of Biomedical Engineering, State University of New York (SUNY), Binghamton, NY 13902, USA; sushilpokhrel@binghamton.edu; 14 College of Korean Medicine, Kyung Hee University, Kyungheedae-ro 26, Dongdaemun-gu, Seoul 05254, Korea; wlsrb7330@khu.ac.kr; 15 Department of Biological Sciences, Faculty of Science, King Abdul-Aziz University (KAU), Jeddah 21589, Saudi Arabia; 16 UKM Medical Molecular Biology Institute, Universiti Kebangsaan Malaysia, Kuala Lumpur 56000, Malaysia

**Keywords:** Merkel cell polyomavirus, Merkel cell carcinomas, drug design, molecular docking, ADMET, MD simulation

## Abstract

Merkel cell carcinoma (MCC) is a rare form of aggressive skin cancer mainly caused by Merkel cell polyomavirus (MCPyV). Most MCC tumors express MCPyV large T (LT) antigens and play an important role in the growth-promoting activities of oncoproteins. Truncated LT promotes tumorigenicity as well as host cell proliferation by activating the viral replication machinery, and inhibition of this protein in humans drastically lowers cellular growth linked to the corresponding cancer. Our study was designed with the aim of identifying small molecular-like natural antiviral candidates that are able to inhibit the proliferation of malignant tumors, especially those that are aggressive, by blocking the activity of viral LT protein. To identify potential compounds against the target protein, a computational drug design including molecular docking, ADME (absorption, distribution, metabolism, and excretion), toxicity, molecular dynamics (MD) simulation, and molecular mechanics generalized Born surface area (MM-GBSA) approaches were applied in this study. Initially, a total of 2190 phytochemicals isolated from 104 medicinal plants were screened using the molecular docking simulation method, resulting in the identification of the top five compounds having the highest binding energy, ranging between −6.5 and −7.6 kcal/mol. The effectiveness and safety of the selected compounds were evaluated based on ADME and toxicity features. A 250 ns MD simulation confirmed the stability of the selected compounds bind to the active site (AS) of the target protein. Additionally, MM-GBSA analysis was used to determine the high values of binding free energy (ΔG bind) of the compounds binding to the target protein. The five compounds identified by computational approaches, Paulownin (CID: 3084131), Actaealactone (CID: 11537736), Epigallocatechin 3-O-cinnamate (CID: 21629801), Cirsilineol (CID: 162464), and Lycoricidine (CID: 73065), can be used in therapy as lead compounds to combat MCPyV-related cancer. However, further wet laboratory investigations are required to evaluate the activity of the drugs against the virus.

## 1. Introduction

Human polyomaviruses are a broad community of human pathogens that normally induce asymptomatic infection in healthy people [1]. The polyomavirus family, which includes the Trichodysplasia spinulosa polyomavirus (TSPyV), John cunningham polyomavirus (JCPyV), BK polyomavirus (BKPyV), and MCPyV, are linked to the growth of various malignant tumors. However, of these, current research only supports the involvement of MCPyV in human carcinogenicity [2,3]. MCPyV is a tiny, non-enveloped, circular double-strand DNA virus that has gained the most attention due to its link with a rare human cancer [4]. The virus is a member of the *Polyomaviridae* family that was first isolated from MCC in 2008 by a group of researchers from the University of Pittsburgh [4,5,6]. Research from diverse geographic regions indicates that approximately 80% of MCC cases occur through MCPyV [6]. MCC is an aggressive neuroendocrine skin cancer linked with immunosuppression induced by MCPyV DNA [6], which has a five-year average survival rate of 40% [7]. The disease is more prevalent in those suffering from leukemia [8] or HIV infection [9] as well as in immunocompromised persons that have undergone organ transplantation [10]. The disease has a mortality rate of 30%, making it more lethal than other cancers, and the incidence of MCC has increased almost four-fold over the past 20 years in the USA [2]. To date, there are no specific therapeutics or vaccines available against the disease.

**Figure 1:**
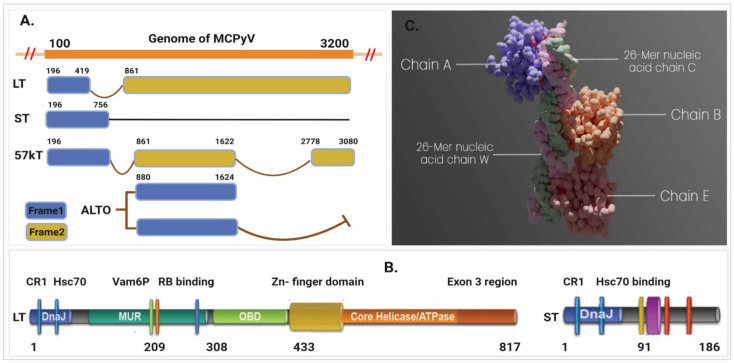
Representing the early region of MCPyV that contains the T (tumor) antigen genome structure. (**A**) The four unique gene products known as the large T (LT), small T (ST), 57kT, and ALTO expressed in the early coding region. (**B**) The large T antigens (left) and small T antigens (right). (**C**) Asymmetric assembly of MCPyV LT antigen origin-binding domains in complex with viral origin DNA retrieved from PDB ID: 3QFQ, adapted from ref. [14].

MCPyV can replicate its own DNA by using the host cell replication machinery [11]. The 5000 base pairs (bp) of the MCPyV closed-circular viral genome can be split up into three regions, namely the viral regulatory, early coding, and late coding regions [6]. The early coding region expresses four unique gene products known as the large T (LT), small T (ST), and 57kT antigens as well as the overprinting gene known as the Alternate frame of the Large T Open reading frame (ALTO), shown in Figure 1 [4,12]. These genes are transcribed before viral DNA replication and express the tumor (T) antigen in the host cell [6]. MCPyV LT antigen is a multifunctional protein that contains several common motifs and domains important for facilitating the viral life cycle [4]. The N-terminal end of MCPyV LT (1–70) amino acids (AA) contains a DnaJ domain comprising conserved region 1 (CR1) followed by the HPDKGG hexapeptide sequence responsible for Hsc70 binding. LT encodes a MCPyV unique region (MUR) binding motif that interacts with vacuolar sorting protein Vam6p in regulating LT stability via multiple E3 ligase interactions (Figure 1). It also contains a LXCXE motif between the first exon and the OBD (~100–300 aa), a stretch of sequences that interferes with retinoblastoma protein (RB) and promotes host cell proliferation [13]. The LT C-terminal region includes an Ori-binding domain (OBD), required for LT to bind to viral Ori, and a helicase domain that stimulates viral genome replication [4]. The majority of MCPyV LT is truncated at 258 aa and thus loses its C-terminal domain responsible for viral replication, whereas the N-terminal contains the RB-interacting domain and promotes cancer development [4,11]. Thus, the integrated virus LT amino terminus plays an important role in virus replication and cancer progression. Therefore, inhibition of the virus LT protein will hinder the replication process as well as the development of MCC.

Plant secondary metabolites and derivatives have gained an abundance of new therapeutic applications in the last century, particularly when applied against cancer [15,16,17]. For instance, plants have aided in the discovery or development of over 60% of anticancer products, either directly or indirectly [18,19]. Previously, numerous plant species and their active components functioning against different diseases have been assessed and shown enhanced activity against different viral and cancer-related diseases [20,21,22]. Natural compounds and their derivatives account for more than half of all FDA-approved medications [18]. However, the identification of molecules that could be promising candidates for drug development is not an easy task because various screening paradigms are required to identify hit molecules [23,24]. For example, high-throughput screening (HTS) involves the screening of an entire compound library directly against the drug target or in a more complex assay system, which is costly and time consuming. In this regard, in silico approaches can be beneficial for discovering new plant-based drugs since they allow the faster completion of a variety of complex tasks, including library screening, drug target prediction, and binding site prediction [21,25]. Computer-aided drug design (CADD) approaches increase the probabilities of recognizing molecules with desirable properties and hastened hit-to-lead development [26,27]. Moreover, new methodology incorporating structure-based drug design with the assistance of informatics tools and analytical approaches has significantly accelerated the drug development process [28,29]. Regarding traditional drug discovery, large-scale in vitro and in vivo trials are needed to calculate a compound’s binding efficacy and toxicity [30]. In this regard, CADD approaches comprise a molecular docking method that can initially screen many compounds with higher binding efficacy. The approaches can also be used to evaluate pharmacokinetic (PK) properties, bioavailability, toxicity, and efficacy of a compound within short periods [31,32]. Additionally, CADD approaches can predict the binding stability of a ligand to its receptor through molecular dynamics (MD) simulation approaches, which is more appropriate [33,34]. Therefore, the goal of this research was to utilize CADD approaches, including molecular docking, pharmacoinformatic, MD simulation, and MM-GBSA methods, to identify bioactive natural compounds that can be used against MCPyV and thereby help in the fight against MCC.

## 2. Results

### 2.1. Protein Preparation

The structure determined for the large T-antigen protein (PDB ID: 3QFQ) with a resolution of 2.90 Å consists of 440 amino acids with a standardized weight of 62.91 kDa, represented in Figure 1C [14]. The asymmetric assembly of MCPyV LT antigen origin-binding domains have three (A, B, and E) identical LT antigen chain in complex with viral origin DNA (C and W) (Figure S1). As the A, B, and E chain are functionally similar proteins that have a similar and identical amino acid (AA) sequence, therefore the B, C, E, and W chains were removed, and A chain was kept for molecular docking simulation. The structure was further modified through two steps in the protein preparation process. In the first step, the metal ions, cofactors, and water beyond 5 Å from the groups were removed, nonpolar hydrogen (H) was merged, and polar H atoms were assigned and saved in PDB file format for further use (Figure S1A). In the second step, the protein was prepared without removing the water and metal ion because water and metal ions sometimes play an important role in the accuracy of ligand–protein docking predictions. As the selected protein crystal structures (3QFQ) contain metal ions and water molecules in their binding sites, the protein was therefore saved with water molecules to determine their role in protein–ligand binding activity (Figure S1B).

### 2.2. *Phytochemical Retrieval and Preparation*

The IMPPAT database was used to select the readily accessible compounds of the desired medicinal plants [24]. A total of 2190 phytochemicals were identified from 104 medicinal plants. The medicinal plants and their corresponding compounds used in this study are listed in Supplementary Tables S1 and S7 (Excel), respectively. The compounds were retrieved from the IMPPAT database and saved in 3D SDF format. Medicinal plants were selected based on a literature review, and their corresponding compounds were retrieved to observe their binding activity toward the MCPyV LT proteins.

### 2.3. *Active Site Identification and Receptor Grid Generation*

**Figure 2:**
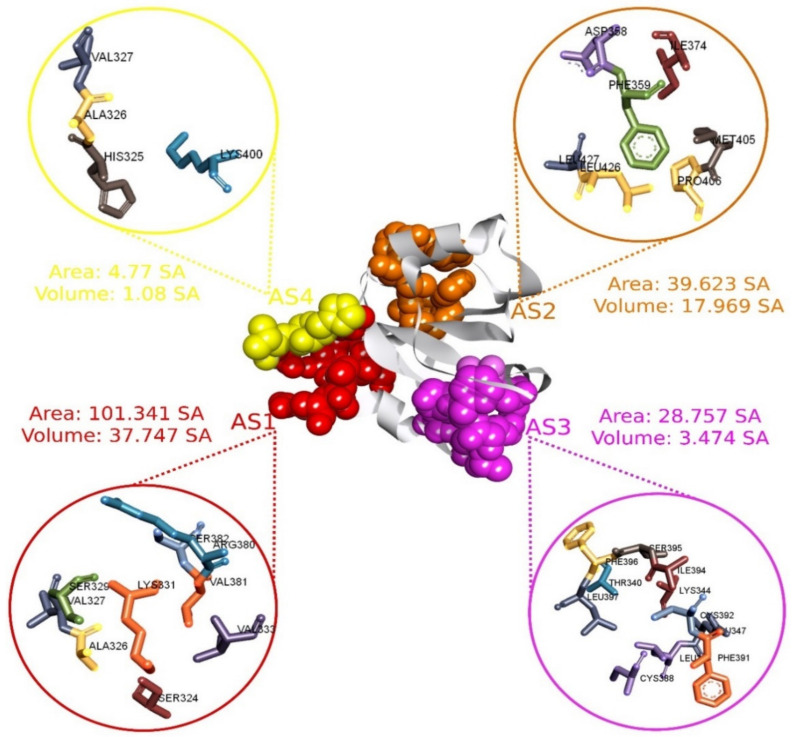
The four selected active pockets of MCPyV LT (PDB: 3QFQ) with the surface area calculated by the CASTp server, adapted from ref. [14]. The first active site (AS1) and its corresponding aa are represented in red, AS2 in orange, AS3 in purple, and AS4 in yellow.

The CASTp server was used to predict the location of the active pocket as well as the binding sites of the MCPyV LT protein [35]. The CASTp web-based tool identified 19 different surface pockets while the probe radius was set to 1.4 Å (Figure S2 and Table S2). Predicted surface pockets were sorted according to area and volume. Among the 19 active pockets, the first 4 active pockets and their corresponding aa residues were chosen based on the surface area and volume listed in Table S3. The first four pockets have a significant area coverage ranging from 103.342 to 4.777 ${Å}^{2}$, where volumes of ≥1 (SA) are shown in Figure 2. The AS residues identified through this evaluation process were utilized for molecular docking purposes. Maximization of receptor active pocket was confirmed through receptor grid box selection process. The prepared protein was imported in PyRx to assign a grid box that generated a box having a center of X = 38.74, Y = −56.48, and Z = −35.11 along with dimensions of X = 30.90, Y = 26.5, and Z = 26.46 Å.

### 2.4. Molecular Docking Analysis

PyRx tools, specifically the AutoDock Vina wizard, were used to perform molecular docking between 2190 phytochemicals and the target protein [36]. Initially, the compounds were docked with protein that does not contain any water molecules in its structure. The docking study found that the binding affinity of compounds ranged between −3.1 and −7.6 kcal/mol. Based on the binding affinity of the five compounds with the highest values, as listed in Table 1, a threshold energy value of ≥−6.50 kcal/mol was chosen for further evaluation. The five selected compounds were subsequently docked with the same protein chain now containing water molecules in the crystal structure. The docking was performed to see how water affected the compound’s binding affinity. Analysis of the docking results show that the presence of water molecules did not substantially alter the binding affinities of the compounds (Table S4).

### 2.5. Interpretations of Protein–Ligand Interactions

Protein–ligand interaction studies are important for understanding the mechanisms of biological regulation, and they provide a theoretical basis for the design and discovery of new drug. They can be categorized into four types: hydrogen bonds, hydrophobic, ionic, and water Bridges. Each interaction type contains more specific subtypes. The interactions are essential to developing novel drug leads, predicting side-effects of approved drugs and candidates, and de-orphaning phenotypic hits [37]. Therefore, the interaction between the five ligands and the target protein was visualized using the BIOVIA Discovery Studio Visualizer software [30]. Interactions between the selected compounds and target protein (with and without water) were also analyzed to observe the different interactions formed in the presence and absence of water and represented in Figure S3.

**Figure 3:**
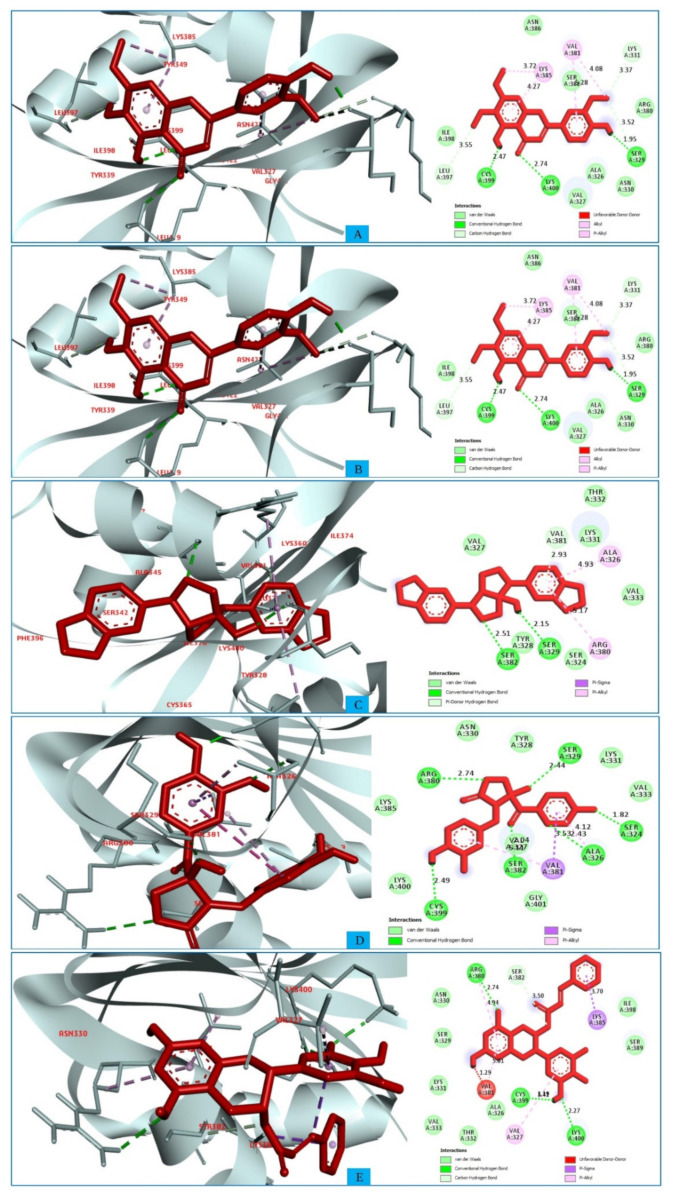
The interactions between the MCPyV large T antigen and five selected natural compounds. The protein–ligand interactions are represented in 3D on the left side of the figure and in 2D on the right side of the figure. The represented interactions are between the MCPyV LT protein and the compounds (**A**) CID: 162464, (**B**) CID: 73065, (**C**) CID: 3084131, (**D**) CID: 11537736, and (**E**) CID: 21629801.

It has been observed that the compound CID: 162464 forms numerous conventional and carbon–hydrogen bonds with the target MCPyV LT protein. It was also found that three conventional hydrogen bonds form at the residue positions SER329 (1.95 Å), LYS400 (2.74 Å), and CYS399 (2.47 Å). Two carbon–hydrogen bonds with LYS331 (3.37 Å) and LEU397 (3.55 Å) were also observed during the interaction of the compound CID: 162464 with the protein. Alkyl and pi-Alkyl bonds were found to form at positions LYS385 (3.72 Å) and VAL381 (4.08 Å) and positions LYS385 (4.72 Å) and VAL381 (5.28 Å), respectively, as shown in Figure 3A and Table S5.

In the case of compound CID: 73065, it was discovered that five conventional hydrogen bonds form at residue positions VAL327 (1.87 Å), SER329 (2.21 Å), SER329 (2.93 Å), SER382 (2.27 Å), and VAL381 (2.67 Å). One pi-alkyl bond was found to form at position VAL381 (4.96 Å) shown in Figure 3B and Table S5. The compound CID: 3084131 generated two conventional hydrogen bonds and one pi-donor hydrogen bond. Two conventional hydrogen bonds were formed at residue positions SER329 (2.15 Å) and SER324 (2.97Å), whereas one pi-donor hydrogen bond was observed at VAL381 (3.81 Å). One pi-sigma bond formed at VAL381 (3.52 Å), and two pi-alkyl bonds formed at ALA326 (4.93 Å) and ARG380 (5.17 Å), as shown in Figure 3C and Table S5. The compound CID: 11537736 was found to form six conventional hydrogen bonds with the target molecule, at positions SER324 (1.82 Å), ALA326 (2.43 Å), SER329 (2.44 Å), ARG380 (2.74 Å), SER382 (2.04 Å), and CYS399 (2.48 Å). One pi-sigma bond formed at VAL381 (3.53 Å), and two pi-alkyl bonds were generated at ALA326 (4.11 Å) and VAL381 (5.10 Å), as shown in Figure 3D and Table S5. For the compound CID: 21629801, five conventional hydrogen bonds and two carbon–hydrogen bonds, and a pi-donor hydrogen bond with the target protein were identified. Five conventional hydrogen bonds at the positions of ARG380 (2.35 Å), VAL381 (2.95 Å), SER382 (2.07 Å), ASN330 (2.05 Å), and CYS399 (2.84 Å) were observed, while two carbon–hydrogen bonds formed at the positions of SER382 (3.21 Å) and SER382 (3.22 Å). One pi-donor hydrogen bond was established at SER324 (2.94 Å), as shown in Figure 3E and Table S5.

### 2.6. Pharmacokinetics (PK) Properties Analysis

PK is a field of pharmacology that employs statistical models to explain and forecast the time course of drug concentrations in body fluids [38]. PK analysis in drug development helps to optimize the absorption, distribution, metabolism, and excretion (ADME) properties of lead compounds. Early PK analysis also helps to develop a therapeutic candidate with an appropriate concentration–time profile in the body for the optimal effectiveness and protection profile [39]. The SwissADME server generates a set of parameters that characterize the drug’s kinetic activity in the body after administration [40]. The tool allows researchers to save time when making informed decisions about the nature and course of studying a molecule’s PK [38]. Therefore, the tool was used to predict the PK properties of the selected molecular candidates. Pharmacological, physicochemical, and PK properties including lipophilicity, solubility, gastrointestinal (GI) absorption, blood–brain barrier (BBB) penetration, and synthesis accessibility of the selected molecules were evaluated and are listed in Table 2 and depicted in Figures S4 and S5.

### 2.7. Toxicity Prediction

An in silico toxicity analysis was performed using the pkCSM web portal to identify the toxic effects of the selected compounds [32]. Oral rat chronic toxicity (LOAEL), oral rat acute toxicity (LD50), AMES toxicity, hepatotoxicity, and skin sensitization of drug candidates were evaluated by the pkCSM server and are listed in Table 3.

### 2.8. *MD Simulation Analysis*

The binding stability of protein–ligand complexes was investigated and validated by using molecular dynamics (MD) simulations. Data focusing on intermolecular interaction were recorded by the MD simulation throughout the orientation time. The study utilized a 250 ns MD simulation to determine the stability of the protein–ligand complexes. The MD simulation findings are reported based on the root mean square deviation (RMSD), root mean square fluctuation (RMSF), intramolecular hydrogen bonding (Intra HB), and protein–ligand contact analysis (P–L contact).

#### 2.8.1. RMSD Analysis

The RMSD is used to quantify the average change in position of a chosen set of atoms relative to a reference atom [41]. The RMSD analysis is used to describe the system equilibration in terms of stability and reliability.

The smaller range of RMSD and constant fluctuation throughout the simulation imply that the protein backbone is stable. On the other hand, a larger RMSD and/or significant variation from the native structure suggest that the protein–ligand combination is more unstable [42]. The mean or average value change between a specific frame and a reference frame with a range order of 1–3 Å is entirely permissible, where a value larger than the required range indicates that the protein has undergone a significant conformational shift. The MD simulation with a time step of 250 ns was used to provide the RMSD that was calculated from Equation (1) and described below.

**Figure 4:**
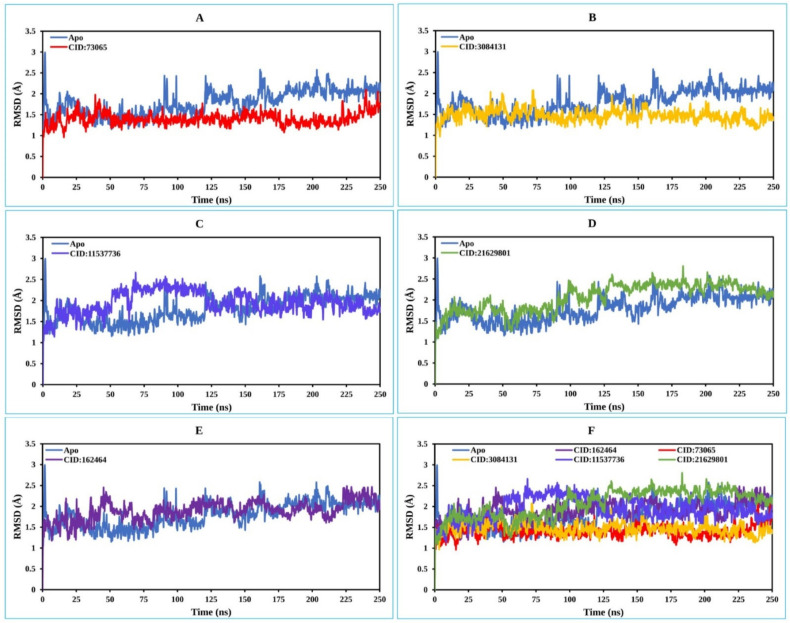
The RMSD values extracted for the Cα atoms of the five selected compounds in complex with the MCPyV LT protein (blue) for the compounds (**A**) CID: 73065 (red), (**B**) CID: 3084131 (yellow), (**C**) CID: 11537736 (light blue), (**D**) CID: 21629801 (green), and (**E**) CID: 162464 (purple), and (**F**) shows all the RMSD for all compounds and the protein together.

Initially, the MCPyV LT protein frames were aligned on the reference frame backbone (blue), and the RMSD of the selected compounds CID: 73065 (red), CID: 3084131 (yellow), and CID: 11537736 (light blue), CID: 21629801 (green), and CID: 162464 (purple) were calculated and are depicted in Figure 4 and Figure S6. The RMSD was computed for the Cα atoms of apoprotein, and the selected compounds increase somewhat but then re-equilibrate toward the conclusion of the 250 ns MD simulation. The RMSD values for the ligand–protein complex was computed and compared with the apoprotein, and the optimal variation was seen for all five molecules. The compound CID: 73065 showed a slight fluctuation between 125 and 175 ns and showed a state of equilibration with the apo structure the rest of the time (Figure 4A). The fluctuations increased for the compound CID: 11537736 from the start of the 25–125 ns MD simulation time and then stabilized with a slight fluctuation between the 166 and 187 ns intervals (Figure 4B). The average RMSD of the compound was 2.067, indicating good stability of the compound with the target protein. The compound CID: 3084131 demonstrates confirmational stability most of the time, having an average RMSD value of 1.25 Å, indicating good stability of the compound with the AS of the protein (Figure 4C). The compound CID: 21629801 exhibits the optimum conformational stability of around 1.2 Å between 200 and 250 ns of the MDS. The highest fluctuation (<2.0 Å) found for the compound is from 50 to 125 ns simulation time, followed by gradual stabilization, indicating that the compound has undergone minor conformational change during the simulation (Figure 4D). In the case of the compound CID: 162464, very low fluctuation was observed during the 250 ns simulation time, although some variations were seen between 25 and 75 ns simulation time, with an average RMSD of 2.5 Å (Figure 4E). Conformational stability was achieved later, from 100 ns and toward the conclusion of the simulation. The measured values for the ligands were less than for the apoprotein RMSD, indicating that the ligand would not dissociate from its original binding site.

#### 2.8.2. RMSF Analysis

**Figure 5:**
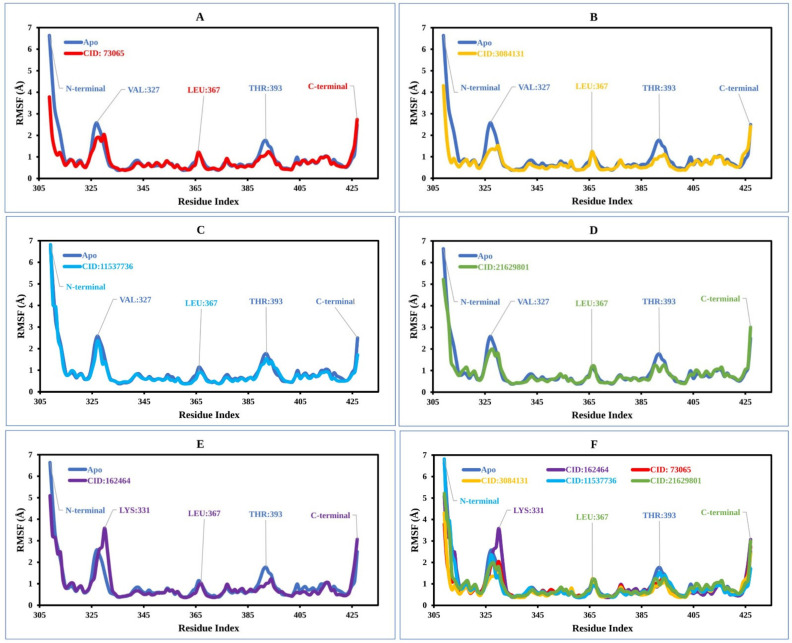
The RMSF values extracted for the Cα atoms of the five selected compounds in complex with the MCPyV LT protein (blue) in complex with the compounds (**A**) CID: 73065 (red), (**B**) CID: 3084131 (yellow), (**C**) CID: 11537736 (light blue), (**D**) CID: 21629801 (green), and (**E**) CID: 162464 (purple), and (**F**) shows the RMSF for all compounds and the protein together.

The root mean square fluctuation (RMSF) can be used to predict the occurrence of local variations in protein chain residues as well as changes in the location of ligand atoms at a given temperature and pressure [43]. Additionally, the RMSF assists in evaluating the flexibility of each atom to get a better understanding of how ligand binding impacts protein flexibility [44]. Low RMSF values of the AA residues suggest that the complex has achieved more stability, while higher values indicate that the complex has achieved less stability [42]. Thus, the RMSF values of the chosen natural compounds CID: 73065 (red), CID: 3084131 (yellow), CID: 11537736 (light blue), (D) CID: 21629801 (green), and CID: 162464 (purple) in combination with the MCPyV LT protein have been analyzed using Equation (2) and are illustrated in Figure 5. In Figure 5, peaks represent the areas of the aa residues that fluctuate the most during the 250 simulations. There was higher fluctuation from the starting to endpoint aa residues of the complex systems than for any other part of the protein due to the location of the N- and C-terminal domains.

In the case of the apoprotein, the maximum fluctuation was observed between residue positions 325 and 330 aa, with a fluctuation of 2.35 Å at VAL327. The apo structure also showed a low level of fluctuation at residue positions LEU367 and THR 393. The apo structure was then compared with the compound CID: 73065 in complex with the protein, in which low fluctuation at residue positions VAL327, LEU367, and THR393 was found (Figure 5A). CID:3084131 seems to have the lowest average RMSF range between 1.0 and 1.3 Å, and the fluctuation of VAL327, LEU367, and THR393 was also low compared to that of the apoprotein structure (Figure 5B). However, the RMSF graph demonstrated average low and significant values of the MCPyV LT protein in complex with CID: 11537736 (0.98 to 1.01 Å), CID: 21629801 (1–1.3 Å), and CID: 162464 (1–1.4 Å) compared to the reference apo structure, as shown in Figure 5C–F. As previously stated, a low RMSF value indicates higher protein stability, whereas the RMSF values found for each protein–ligand system in this study were lower than those for apoprotein. Therefore, the compounds are expected to retain stable contact with the protein without altering its structure.

#### 2.8.3. Protein–Ligand Contacts

**Figure 6:**
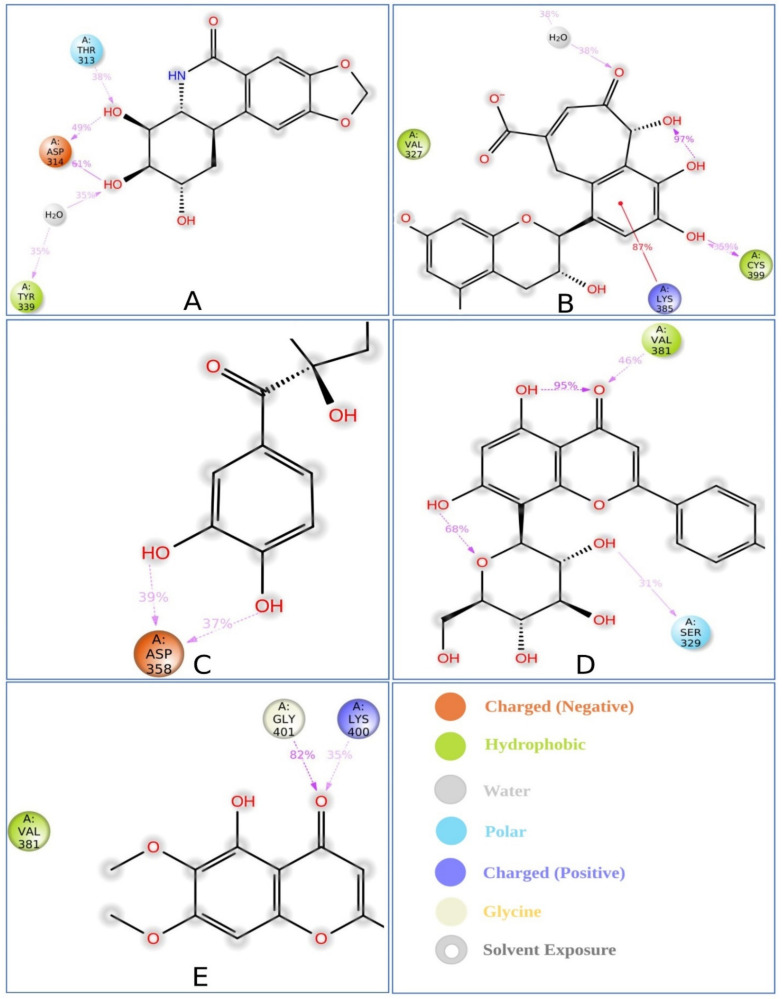
Schematic representation of interactions of selected ligand atoms with MCPyV LT protein residues shown for interactions that occur more than 30.0% of the simulation time between the protein and the compounds (**A**) P CID: 73065, (**B**) CID: 3084131, (**C**) CID: 11537736, (**D**) CID: 21629801, and (**E**) CID: 162464 in the selected trajectory (0.00 through 250.00 ns).

The bonding interaction between the molecules and the target protein plays an important role in the stability as well as PK properties. For example, hydrogen bonds of molecules affect drug selectivity, adsorption, and metabolism. Therefore, the protein complex with the selected ligands and their intermolecular interactions were studied using a simulation interactions diagram (SID). The interactions that occur for more than 30.0% of the simulation time between the natural compound (CID: 73065, CID: 3084131, CID: 11537736, CID: 21629801, and CID: 162464) atoms and the MCPyV LT protein residues were characterized based on hydrogen, hydrophobic, ionic, and water bridge bonds and are represented in Figure 6. Additionally, a stacked bar chart representation of the protein–ligand interactions found during the 250 ns simulation run is also provided in Figure S7. The interaction between the protein and ligands can be described based on an interaction fraction value (IFV), such as that an IFV value of 0.7 suggests that the specific interaction is maintained for 70% of the simulation time. Values over 1.0 (>100%) are possible, as some protein residues may make multiple contacts of the same subtype with the ligand. For example, the ARG side chain has four H-bond donors that can produce four hydrogen bond interactions with a single H-bond acceptor.

In this study, atoms of the compound CID: 73065 formed interactions at ASP314 and TYR339 protein residues for more than 30.0% of the simulation time with an IFV of 1.4 and 0.6, respectively, indicating that the specific interaction was maintained for 140% and 60% of simulation time via multiple contacts of the same subtype in the ligand (Figure 6A and Figure S6A). The compound CID: 3084131 produced multiple contacts at LYS385, ASN386, and CYS399 aa residues with 2.0, 0.75, and 1.2 IFV, respectively (Figure 6B and Figure S6B), where the compound CID: 11537736 formed multiple contacts with ASP358 residue with an IFV of 1.2 (Figure 6C and Figure S6C). In the case of the compound CID: 21629801, contact occurred at SER329 and VAL381 residues for more than 30.0% of the simulation time with IFVs of 0.78 and 0.65, respectively (Figure 6D and Figure S6D). Finally, the compound CID: 162464 was found to form multiple interactions for more than 30.0% of the simulation time at the residue positions LYS400 and GLY401 with an IFV of 0.88 and 0.8, respectively, suggesting that the specific interaction will be maintained by multiple contacts for 88% and 80% of the simulation time, respectively, and help to ensure stable binding with the target protein (Figure 6E and Figure S6E).

### 2.9. MM-GBSA Analysis

**Figure 7:**
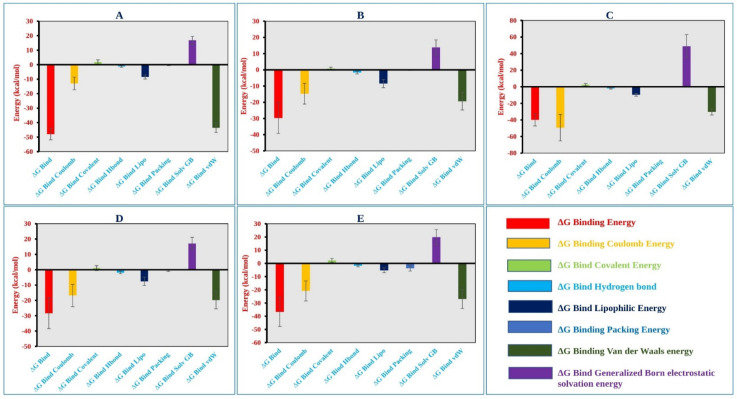
Different energy components and net MM-GBSA binding free energy (kcal/mol) along with the standard deviation values calculated from 250 ns MD simulation trajectory of MCPyV LT protein in complex with the selected compounds (**A**) CID: 162464, (**B**) CID: 73065, (**C**) CID: 3084131, (**D**) CID: 11537736, and (**E**) CID: 21629801.

The MM-GBSA approach helps to determine the binding free energy of a molecule to the target protein. The binding free energy of the selected molecules to the target protein were evaluated based on the MD simulation trajectory and are represented in Figure 7. The MM-GBSA of the complex structure was computed for every single frame generated for the 250 ns MD simulation trajectory. The analysis of the complex structure identified higher net negative binding free energy values of −47.91 ± 3.92, −29.67 ± 9.53, −39.98 ± 7.20, −28.34 ± 10.05, and −36.67 ± 11.0 kcal/mol for the five selected molecules CID: 162464, CID: 73065, CID: 3084131, CID: 11537736, and CID: 21629801, respectively, with the target protein (Figure 7).

In addition, the physicochemical contributions to total energy from the analysis of different components found a significant contribution of coulomb, covalent, van der Waals, lipophilic, and generalized Born electrostatic solvation energy shown in Figure 7 and listed in Table S6. Therefore, it can be said that the five selected molecules will maintain a stable interaction with the target protein.

## 3. Discussion

MCC is a very rare disease in which malignant (cancer) cells form on the top layer of the skin. MCPyV is responsible for at least 80% of all cases of MCC, which has a high risk of returning (recurring) and spreading (metastasizing) [4,45,46,47]. MCC has a mortality rate of 30%, and to date, no specific drugs are available against the disease [11]. Therefore, the study aimed to identify effective drug candidates against the disease using CADD approaches. CADD approaches have proven efficient and successful in the field of drug design and development, as they enable researchers to rapidly identify the most successful drug candidates. It has been found that CADD approaches can reduce the cost of the drug development process by up to 50% [48]. These approaches can be used to search for new compounds based on specific targets and, in the screening process, increase the possible number of compounds evaluated in a short time. CADD approaches include virtual scanning, molecular docking, ADMET, and MD simulation and are commonly used to find, produce, and study drugs and related bioactive molecules [30].

Molecular docking is a popular method used in the rational design of a drug that involves the analysis and prediction of binding behaviors and interaction affinities between ligands and receptors [49,50]. To identify the interaction between the desired target with different natural compounds in this study, a molecular docking-based screening process was initially applied. This study conducted the simulated screening of a vast library of natural phytochemicals that could target the MCPyV LT protein. The docking technique efficiently screened and ranked 2190 phytochemicals based on a scoring function, whereby the top five phytochemicals were selected based on having the highest binding affinity. The five selected compounds CID: 73065, CID: 11537736, CID: 21629801, CID: 162464, and CID: 3084131 had the highest binding scores of −6.6, −6.7, −7.1, −6.5, and −7.6 kcal/mol, respectively. The selected compounds were further evaluated based on their PK properties.

PK is the study of how small drug-like molecules are absorbed, distributed, metabolized, and excreted (ADME) by the body [39]. Since ADME processes influence the intensity and duration of a drug’s operation, their interpretation is critical for supervising the drug development phase and clinical decision-making. Understanding a drug’s absorption and distribution properties helps us to estimate how much of an administered dosage can penetrate the bloodstream and hit the target site of action. Furthermore, knowledge of drug metabolism and elimination accounts for the prediction of concentrations for drugs that are provided regularly [51]. Therefore, the study also evaluated a drug’s effectiveness by determining its ADME properties [52]. As a result of pharmacokinetic analysis, the optimal GI absorption of the selected compounds with no BBB permeation was identified. The consensus log Po/w value of candidate molecule ˂5 indicates that it is lipophilic and, therefore, capable of crossing lipid membranes found within the body. Lipinski’s rule of five (LR5) assesses the drug-like properties of chosen compounds to determine if they are orally bioavailable for humans, and the selected compounds were found to have good drug-likeness properties.

In the human body system, biological regulators control a variety of cellular processes, including biosynthesis, signal transduction, transport, storage, and metabolism. The undesired activity of a drug candidate with a bioregulator, excluding the primary target, can show toxic effects [53]. One of the most prominent causes of late-stage drug development failure is drug toxicity [54]. It has been found that 20% of late-stage drug discovery failures are due to molecule toxicity [32]. Recently, high toxicity estimation approaches for the early stages of drug production have evolved, which maximize the success ratio of corresponding drug development phases [54]. Early-stage toxicity of drug candidates can be evaluated through in silico toxicology techniques that utilize both quantitative and qualitative methods. In silico methods have many advantages, including the potential to analyze hypothetical substances, their low expense, and the fact that such simulated studies are usually focused on human results, which eliminate the issue of interspecies transferability [53]. Therefore, the study evaluated the toxicity of the selected drug candidates through computational approaches. The top five phytochemicals were evaluated based on mutagenicity, organ toxicity, and animal-based toxicity analysis. The Ames analysis is a bacterial mutation assay that was used in this study to determine whether the medications may trigger such gene mutations that cause genetic disruptions to a cell that can eventually lead to cancer [55], and showed that all tested compounds were negative. Besides this, they were not found to chemically alter the skin, which may result in a prolonged T-cell-induced allergic reaction [56], nor to trigger hepatic dysfunction [57]. LD50 estimates the short-term toxicity efficiency of compounds over a specific time [54], and all compounds were found to have optimum acute toxicity. Moreover, the chosen phytochemicals exhibited favorable results in chronic toxicity tests that were repeated over a longer duration on the living organism.

The compounds that have been chosen based on docking, PK, and toxicity properties were further evaluated through MD simulation methods. MD simulation was used to identify the binding stability of the selected compounds to the AS of the target protein. MD simulations help to determine the physical motions of molecules inside a desirable macromolecule and have become an integral part of the CADD process [58]. MD simulations enable analyzing an intended drug candidate’s stability in the presence of a certain macromolecule. Therefore, the LT protein in complex with the five selected small molecules was evaluated based on the RMSD, RMSF, and ligand–protein interactions generated from the 250 ns simulation trajectories. Optimal RMSD and RMSF values for all five compounds were obtained, and the protein–ligand interactions indicated the stability of the five selected molecules to the AS of the protein. Additionally, in the MM-GBSA calculated from the simulation trajectory, a high ΔGbind value was found, as well as coulomb, covalent, vdw, lipophilic, and solv gb energy, indicating the stability of the selected compounds to the target protein for long-term simulation.

The comprehensive CADD approaches successfully identified five compounds, namely Lycoricidine (CID: 73065), Actaealactone (CID: 11537736), Epigallocatechin 3-O-cinnamate (CID: 21629801), Cirsilineol (CID: 162464), and Paulownin (CID: 3084131), which have previously shown activity against different diseases. For example, Lycoricidine, a type of amaryllidaceae alkaloid and constituent of the medicinal plant *Lycoris radiata,* which has shown anti-HCV activity by downregulating the expression of host heat-stress cognate 70 (Hsc70) [59]. Paulownin, found in *Gmelina arborea*, has recently been reported to have a potential inhibitory effect against key receptors of estrogen-positive breast cancer, including FGFR2, ESR1, PIK3CA, PIK3CB, and PIK3CD [60]. The compound has also been reported to have anti-inflammatory and analgesic properties and boost immunity as well as lower blood glucose levels [61]. Actaealactone, a type of neolignan found in the extracts of *Actaea racemose,* is reported to have antioxidant activity [62]. On the other hand, Cirsilineol, a flavone from *Artemisia capillaris*, has many medicinal properties such as anti-neoplastic activity [63], induction of ROS-mediated apoptosis [64], and inhibition of IFN-g signaling in a murine model [65]. The role of the compound Epigallocatechin 3-O-cinnamatein in different diseases, however, has not yet been studied. The study here identified potential inhibitory compounds that may act against the MCPyV LT protein. Previously, most studies were designed to find a potential lead compound by analyzing a plethora of similar compounds. In this study, however, we have analyzed various compounds that cover a wide array of natural compounds, enhancing the chance of discovering a promising drug candidate against MCPyV LT antigen.

## 4. Materials and Methods

### 4.1. *Protein Preparation*

The three-dimensional (3D) structure of the MCPyV large T antigen protein was obtained from the Protein Data Bank (www.rcsb.org//pdb, accessed on 9 December 2021) and has previously been generated via X-ray diffraction. PyMOL v2.4.1 was chosen to view the PDB configuration of the receptor, which was prepared for further analysis using Schrödinger’s Protein Preparation Wizard [66].

### 4.2. *Compound Retrieval and Preparation*

Indian Medicinal Plants, Phytochemistry, And Therapeutics (IMPPAT) (https://cb.imsc.res.in/imppat/home) (accessed on 9 December 2021) is a manually compiled database of 1742 Indian Medicinal Plants and 9596 stereochemically diverse bioactive compounds [24]. Phytochemicals from a variety of medicinal plants were retrieved from the database and prepared by applying OPLS_2005 as a force field. The Epik ionization tool of Schrödinger Suite was utilized to obtain the ionization state of the compound, where the pH was set within the range of 7 ± 2 [67]. All possible deprotonated and ionization states along with their tautomers, stereochemistry, and ring conformations of the compound were also determined during the ligand preparation process.

### 4.3. *Active Site Identification and Receptor Grid Generation*

The active site (AS) of a protein or enzyme is a complex structure of different aa residues in a particular region that aid in the formation of a temporary attachment with the substrate [68]. The active sites forming aa residues are also known as the binding site, which enable the enzyme to bind to a chemical substrate and catalyze the reaction. Additionally, it aids in the stabilization of reaction intermediates and helps recognizing the ligand and forming a tight binding relationship with the protein [32]. Identification of the AS position in a protein can help to generate enough contact points with the ligands and significantly increase the docking efficiency. Therefore, the Computed Atlas of Protein Surface Topography (CASTp) 3.0, a web-based tool (http://sts.bioe.uic.edu/castp/) (accessed on 9 December 2021) was used to classify all surface pockets, internal cavities, and cross-channels in our refined protein structured model [35]. The refined 3D protein structure was uploaded to the website to analyze the binding pocket of the MCPyV LT protein. Additionally, the area and volume of the binding pocket as well as the aa residues required for binding interaction were estimated.

### 4.4. *Molecular Docking*

PyRx software was used to upload the refined 3D structure of target proteins and small phytochemical molecules for structure-based virtual screening [36]. Before beginning the simulated screening process, the referenced 3D protein was transformed into a macromolecule, and all phytochemicals were subjected to an energy minimization phase [69]. PyRx virtual scanning tools convert the PDB format of a macromolecule and SDF format of small molecules to Autodock’s Pdbqt format using the Open Babel widget according to default configuration parameters. Afterward, all aa residues were selected for the following process using the CASTp server. The grid box was adjusted using Vina Wizard to guarantee that all chosen amino acid residues are contained inside the grid box for running Vina Wizard. Finally, it analyzes the protein binding free energy, which is associated with the scoring mechanism for deciding which phytochemicals are more likely to attach to the target [49], and the phytochemicals containing the highest binding energy (kcal/mol) with a negative sign were selected for further analysis. Finally, the binding interaction between the protein and ligand complex was visualized using BIOVIA Discovery Studio Visualizer v 20.1.0.19295 (BIOVIA) [70].

### 4.5. *Pharmacokinetic (PK) Properties Analysis*

Pharmacokinetics is the analysis of how drug concentrations change with time in various body fluids in consideration of the drug absorption, distribution, metabolism, and excretion (ADME) properties. Lipinski’s rule specifies the following criteria for an orally active drug: MW (200~500), HBAs (0~10), HBDs (0~5), ClogP ≤ 5, and ROTBs (0~10) [71]. The PK properties of the compounds were evaluated using the SwissADME (http://www.swissadme.ch/index.php) server (accessed on 12 December 2021) [40]. The SwissADME tool predicts the pharmacokinetic, pharmacological, and physicochemical properties, including gastrointestinal (GI) absorption, lipophilicity (consensus Log Po/w), blood–brain barrier (BBB) penetration, solubility (ESOL), drug likeness, and medicinal chemistry friendliness [72].

### 4.6. *Toxicity Prediction*

The pkCSM (http://biosig.unimelb.edu.au/pkcsm/) server (accessed on 13 December 2021) is a freely available web server for developing statistical models of drug discovery using a graph-based signature that assesses the toxicity of a variety of substances to guarantee the clinical effectiveness of drug candidates [73]. The pkCSM web server was used here to predict the toxicity parameters, including AMES toxicity, oral rat acute toxicity, oral rat chronic toxicity, hepatotoxicity, and skin sensitization of the compounds.

### 4.7. MD Simulations

MD simulations were conducted using the Desmond v3.6 program in Schrödinger to determine the stable interactions of the ligands to the binding pockets of the receptors [67]. The stability of the selected compounds in binding the target protein was determined through 250 ns MD simulations. For the prediction of the equation of state (EOS), this software allowed automated simulation and free energy perturbation (FEP) computation, which was combined with various temperatures. The system was solvated using a predefined TIP3P water model, in which an orthorhombic periodic boundary box shape with 10 Å was employed to assign both sides to retain a certain volume. The system was electronically neutralized using suitable ions such as Na^+^ and Cl^−^ with salt concentration of 0.15 M. After creating the solvated system that included the protein in complex with the ligand, the system was optimized by employing the OPLS-2005 force field. Ensembles of NPT (constant number of particles, pressure, and temperature) particles were maintained at 300 K and 1.0 atmospheric (1.01325 bar) pressure, followed by 250 PS recording intervals with an energy of 1.2, in which the solvent and ions were evenly distributed around the protein–ligand complex.

### 4.8. Simulation Trajectory Analysis

The quality of the MD simulation was verified, and the simulation event was analyzed using simulation interaction diagram (SID) available in the Schrödinger package [68]. The stability of the complex structure was assessed based on the RMSD, RMSF, protein–ligand interactions (P–L contacts), and hydrogen bond interactions found from the trajectory.

#### 4.8.1. RMSD Analysis

The RMSD of a protein–ligand complex system enables the average distance produced by the dislocation of a chosen atom over a certain period to be determined and indicates the stability of a protein [45,74]. When initializing the simulation, the protein structures and the reference frame backbone were aligned, and after that, the RMSD of the whole system was computed for about the duration that the MD simulations ran (in our instance, 250 ns). The RMSD of a complicated system with a time of x may be computed using the below equation.

(1) $${\mathrm{RMSD}}_{x}=\sqrt{\frac{1}{N}}\sum_{i=1}^{N}(r_{i}^{\prime }(t_{x}))-r_{i}(t_{\mathrm{ref}}){)}^{2}$$

where *N* is the atom selection number, t_ref_ is the reference time (usually the first frame is a reference and time t = 0), and *x* is the location of the chosen atoms when superimposed with the reference frame. Every simulation frame undergoes the process.

#### 4.8.2. RMSF Analysis

The generated RMSF information plays an important role in identifying the local conformational change of a protein coupled with ligands by calculating the average observed atomic changes compared to the number of atoms [30,32]. The following equation may be used to determine the RMSF value of a protein, where the number of residues is denoted by *i*.

(2) $${\mathrm{RMSF}}_{i}=\sqrt{\frac{1}{T}}\sum_{t=1}^{T}<(r_{i}^{\prime }(t))-r_{i}(t_{\mathrm{ref}}){)}^{2}>$$

where T denotes the trajectory time interval used to calculate the RMSF, t_ref_ denotes the reference time interval, $r_{i}$ denotes the location of residue *i*, $r_{i}^{\prime }$ denotes the location of the atoms in residue *i* after their overlap upon that reference, and the angle brackets (< >) denote that the square distance is averaged across the residue’s atoms.

### 4.9. MM-GBSA Analysis

MM-GBSA is a popular method to calculate the binding free energy of a complex of molecules with a protein or a free ligand [70]. The MM-GBSA of a complex system can be calculated based on MD simulation trajectory, which is more accurate than most scoring functions. Therefore, to determine the binding free energy (ΔGbind) of the selected molecules in complex with MCPyV LT protein, the MM-GBSA methods were utilized through the Prime MM-GBSA module in the Schrödinger Maestro package [67].

## 5. Conclusions

Premature stop codon mutations in the truncated MCPyV LT protein are primarily responsible for the proliferation of cutaneous malignant cells that lead to MCC through Merkel cell transformation [7,11]. However, no effective drugs have been identified that target the protein and may thus help to combat against MCC-related human cancers. Therefore, this study aimed to finding natural and effective chemicals that might restrict the function of protein and, hence, impede cancer growth. To achieve this, the research applied a broad range of computational methods, including molecular docking, ADMET, MD simulation, and MM-GBSA approaches, and identified five promising therapeutic candidates, namely Lycoricidine (CID:73065), Actaealactone (CID:11537736), Epigallocatechin 3-O-cinnamate (CID:21629801), Cirsilineol (CID:162464), and Paulownin (CID:3084131), which can inhibit the MCPyV LT protein activity and subsequently block cancer formation. However, these phytochemicals must be subjected to in vitro and in vivo investigations to evaluate their effectiveness and safety as anti-MCPyV medicines in humans. Developing the selected phytochemicals as drug candidates against MCPyV is both therapeutically and commercially feasible.

## Figures and Tables

**Table 1:** List of compounds, CAS ID, PubChem CID, chemical formula, and two-dimensional (2D) structure of the five selected compounds with the highest binding affinity.

No.	CAS ID	PubChem CID	Chemical Name	Chemical Formula	2D Structure	Docking Score (kcal/mol)
1	13040-46-5	CID: 3084131	Paulownin	C_20_H_18_O_7_	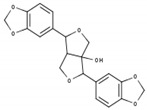	−7.6
2	108907-46-6	CID: 21629801	Epigallocatechin 3-O-cinnamate	C_24_H_20_O_8_	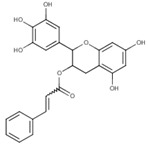	−7.1
3	874359-26-9	CID: 11537736	Actaealactone	C_18_H_14_O_8_	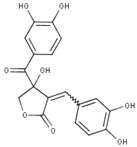	−6.7
4	19622-83-4	CID: 73065	Lycoricidine	C_14_H_13_NO_6_	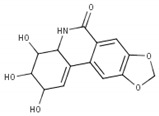	−6.6
5	41365-32-6	CID: 162464	Cirsilineol	C_18_H_16_O_7_	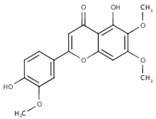	−6.5

**Table 2:** List of pharmacokinetic properties, including ADME properties, of the five selected natural compounds. The list also includes the different physicochemical properties of the compounds.

Properties		CID: 73065	CID: 11537736	CID: 3084131	CID: 21629801	CID: 162464
Physicochemical Properties	Formula	C_14_H_13_NO_6_	C_18_H_14_O_8_	C_20_H_18_O_7_	C_24_H_20_O_8_	C_18_H_16_O_7_
	MW (g/mol)	291.26	358.30	370.35	436.41	344.32
	Heavy atoms	21	26	27	32	25
	Arom. atoms	6	12	12	18	16
	Rotatable bonds	0	3	2	5	4
	H-bond acceptors	6	8	7	8	7
	H-bond donors	4	5	1	5	2
Lipophilicity	C Log Po/w	−0.42	0.78	2.10	2.45	2.53
Water Solubility	Log S (ESOL)	−1.01	−2.9	−3.35	−4.31	−4.33
Pharmacokinetics	GI absorption	High	Moderate	High	Moderate	High
	BBB permeant	No	No	No	No	No
Drug Likeness	RO5 Violation	0	0	0	0	0
Medi. Chemistry	Synth. ability	4.01	3.61	4.22	4.38	3.43

**Table 3:** A list of the drug-induced toxicity profile for the five selected natural compounds.

Target	CID: 73065	CID: 11537736	CID: 3084131	CID: 21629801	CID: 162464
AMES toxicity	No	No	No	No	No
LD_50_	1.981	2.154	2.241	2.769	2.258
LOAEL	2.907	3.172	1.684	3.834	0.953
Hepatotoxicity	No	No	No	No	No
Skin Sensitization	No	No	No	No	No

## Data Availability

Data is contained within the article and Supplementary Material.

## Supplementary Materials

The following are available online at https://www.mdpi.com/article/10.3390/ph15050501/s1. Figure S1: Representing the MCPyV LT protein retrieved from the protein data bank (PDB ID: 3QFQ-A). Herein, (A). Asymmetric assembly of MCPyV LT antigen origin-binding domains in complex with viral origin DNA. (B) The prepared protein chain without the water molecules, and (C) the prepared protein chain with the water molecules. Figure S2: The active pocket of MCPYV LT (PDB: 3QFQ) with surface area and volume calculated by the CASTp server indicated in the red circle. The server generated a total of 19 pockets, but one was excluded from the figure due to the active pocket being nonvisible due to low surface area and volume. Figure S3: The interaction between the MCPyV LT protein and five selected natural compounds. Interaction of the ligands and protein with water are represented in the left side of the figure, and the interaction of ligands and protein without water are depicted in right side of the figure. The interaction is shown between MCPyV LT protein and the compounds (A) CID: 162464, (B) CID: 73065, (C) CID: 3084131, (D) CID: 11537736, and (E) CID: 21629801. Figure S4: Data visualization of amino acid interaction for (A) comparative bond length, (B) bond category, and (C) bond distance. Figure S5: Data visualization of (A) molecular weight, (B) number of heavy atoms, (C) aromatic heavy atoms, (D) rotatable bonds, (E) hydrogen bond acceptors, (F) hydrogen bond donors, (G) lipophilicity, and (H) water solubility of selected molecules. Figure S6: Representing multiple repetition of RMSD value for each 50 ns interval obtained from 250 ns Simulation time. Herein, A. 1–50 ns, B. 50–100 ns, C. 100–150 ns, D. 150–200 ns, and E. 200–250 ns RMSD of the selected protein–ligand’s complex structure. Figure S7: Stacked bar charts representing the protein–ligand interactions found during the 250 ns simulation run for (A) CID: 73065, (B) CID: 3084131, (C) CID: 11537736, (D) CID: 21629801, and (E) CID: 162464 compounds in complex with the MCPyV LT protein. Table S1: List of plants and their antiviral activity against different viruses, and the compounds derived from them are retrieved from the IMPPAT database for this study. Table S2: Active pocket of the MCPyV LT protein (PDB ID: 3QFQ; Chain: A) with surface area and volume. Table S3: First four active pockets and their corresponding aa residues retrieved from the MCPyV LT protein (PDB ID: 3QFQ; Chain: A). Table S4: Comparative docking score of compounds for docking of the protein with water and without water. Table S5: List of bonding interactions between five selected phytochemicals with MCPyV large T antigen. Table S6: The contributions to the total energy from the analysis of different components through MM-GBSA energy calculation method. Analysis of MM-GBSA for five selected compounds found a significant contribution in the binding, coulomb, covalent, van der Waals, lipophilic, and generalized Born electrostatic solvation energy. Table S7: Corresponding compounds of medicinal plants used in this study.
