# Supplementary material for: Toward the Identification of Natural Antiviral Drug Candidates against Merkel Cell Polyomavirus: Computational Drug Design Approaches
Source: Pharmaceuticals (Basel). 2022 Apr 20;15(5):501. doi: 10.3390/ph15050501 (PMC9146542; doi:10.3390/ph15050501)
Supplement: Supplementary file 1 [file pharmaceuticals-15-00501-s001.zip › Supplimentary Figure (F).pdf]

*Supplementary Figure*

**Towards the Identification of Natural Antiviral Drug Candidates Against Merkel Cell Polyomavirus: Computational Drug Design Approaches**

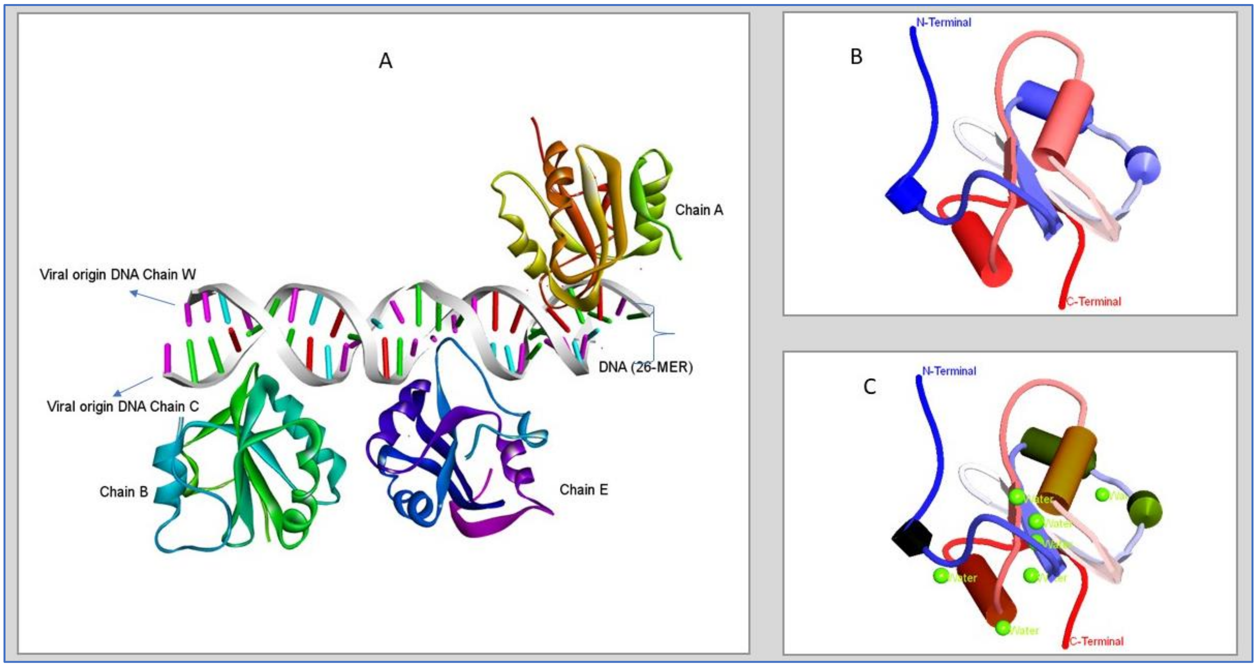


**Figure S1.** Representing the MCPyV LT protein retrieved from the protein data bank (PDB ID: 3QFQ-A). Herein, (A). Asymmetric assembly of MCPyV LT antigen origin-binding domains in complex with viral origin DNA. (B). depicted the prepared protein chain without the water molecules, and (C). the prepared protein chain with the water molecules.

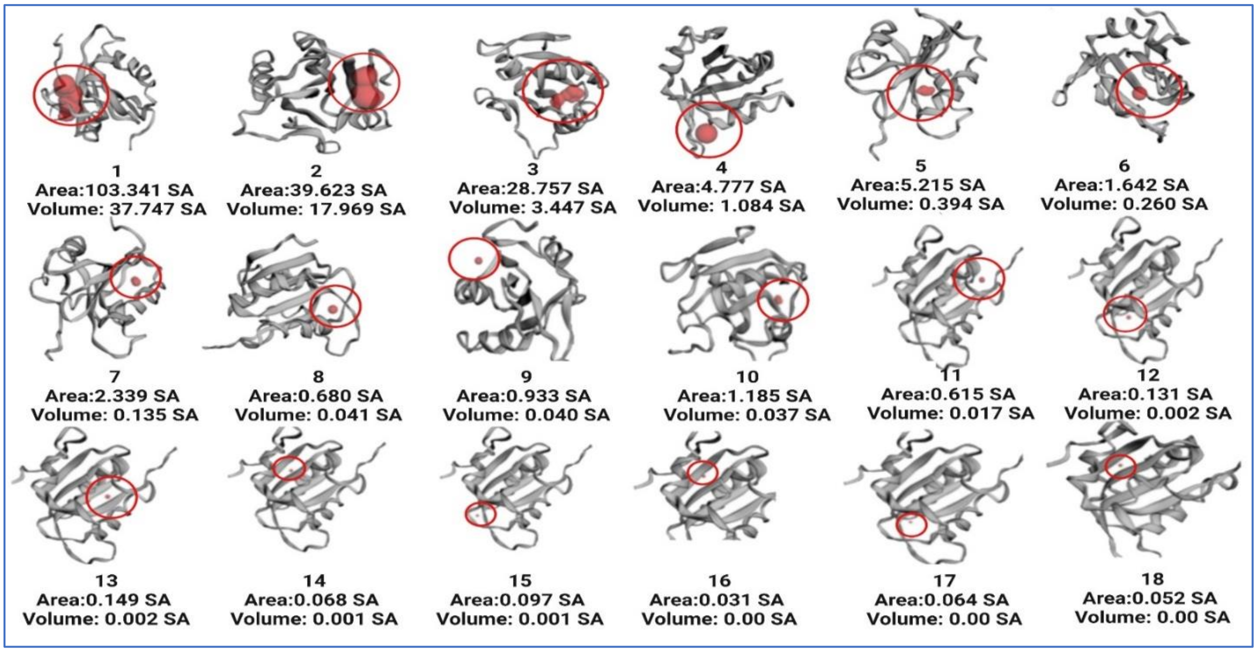

**Figure S2**. Representing the active pocket of MCPYV LT (PDB: 3QFQ) with the surface area and the volume indicated in red round shape calculated by the CASTp server. The server generated a total of 19 pocket, but one pocket has been excluded from the figure due to non-visible active pocket with low surface area and volume.

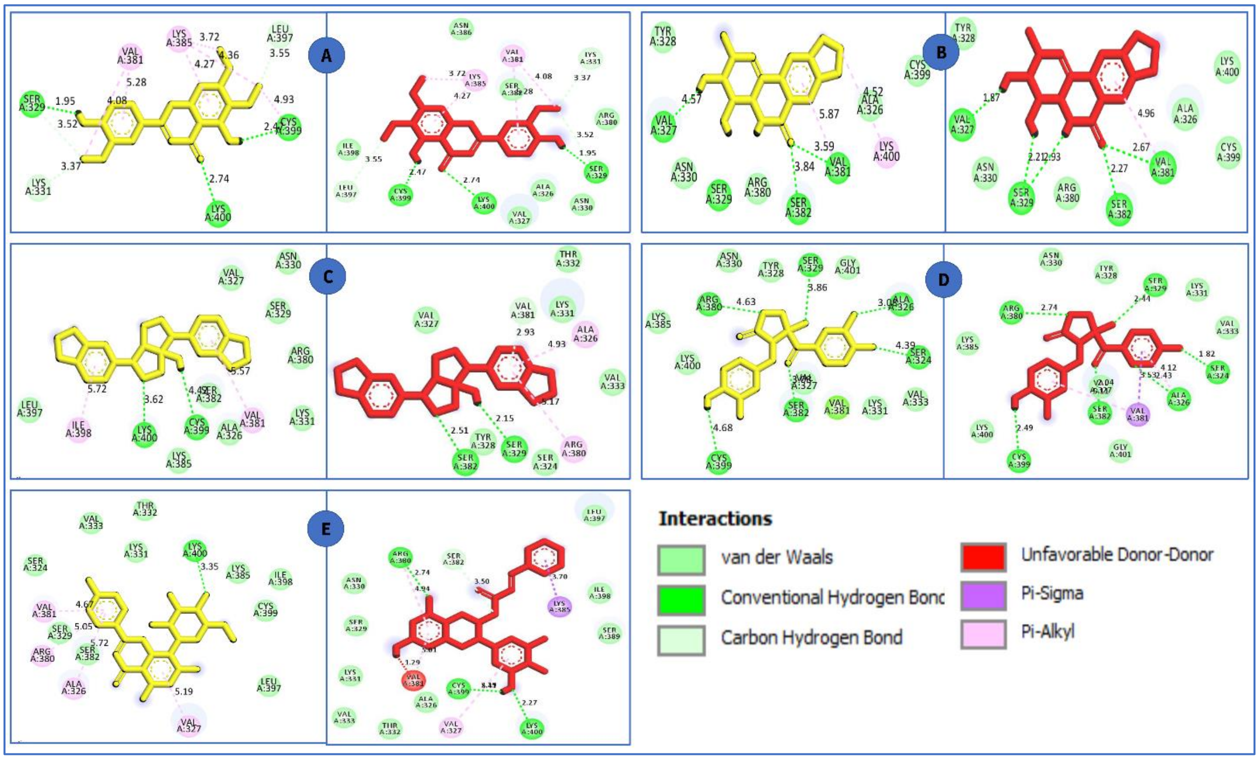


**Figure S3.** The interaction between the MCPyV LT protein and selected five natural compounds. The ligands and protein with water has represented left side in the figure, where ligands and protein without water interaction has depicted in right side of the figure. Herein, representing the interaction between the MCPyV LT protein with the compounds (A). CID:162464, (**B).** CID:73065, (C). CID:3084131, (D). CID:11537736, and (E). CID:21629801.

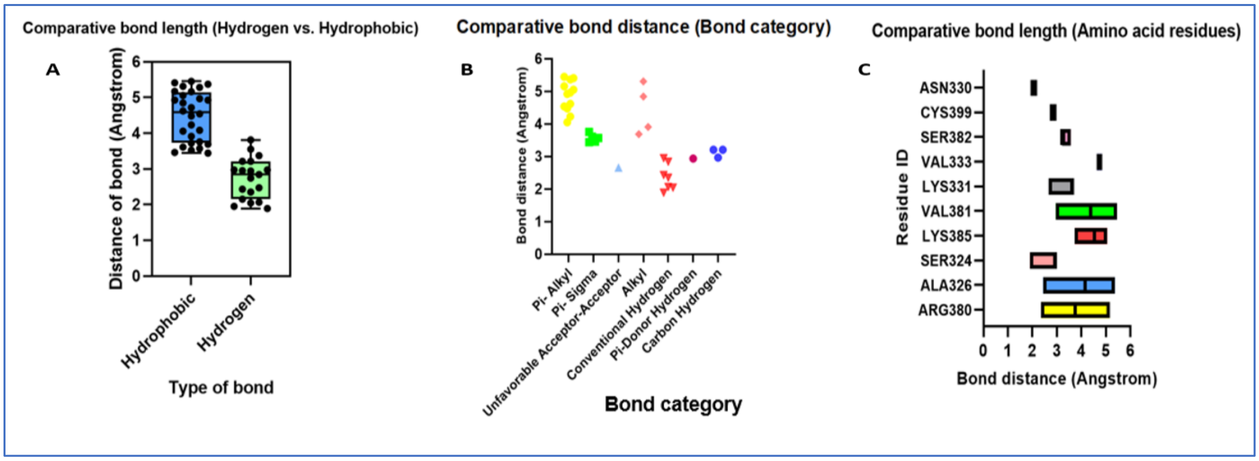


**Figure S4**. Data visualization of amino acid interaction for A) comparative bond length, B) bond category and C) bond distance

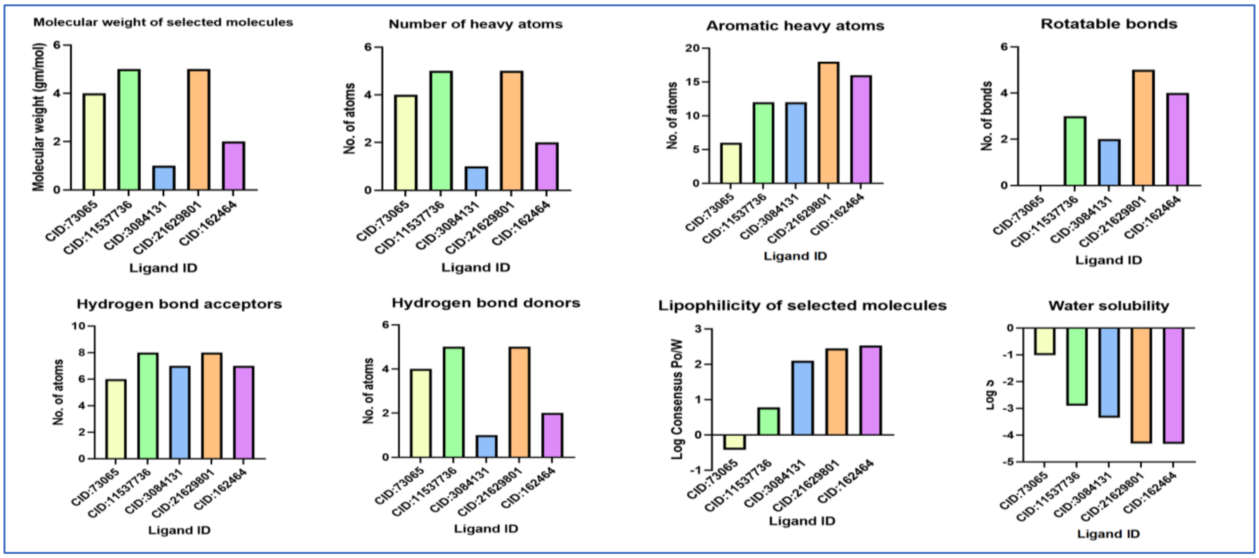


**Figure S5**. Data visualization of A) Molecular weight, B) Number of heavy atoms, C) Aromatic heavy atoms, D) Rotatable bonds, E) Hydrogen bond acceptors, F) Hydrogen bond donors, G) Lipophilicity and H) Water solubility of selected molecules.

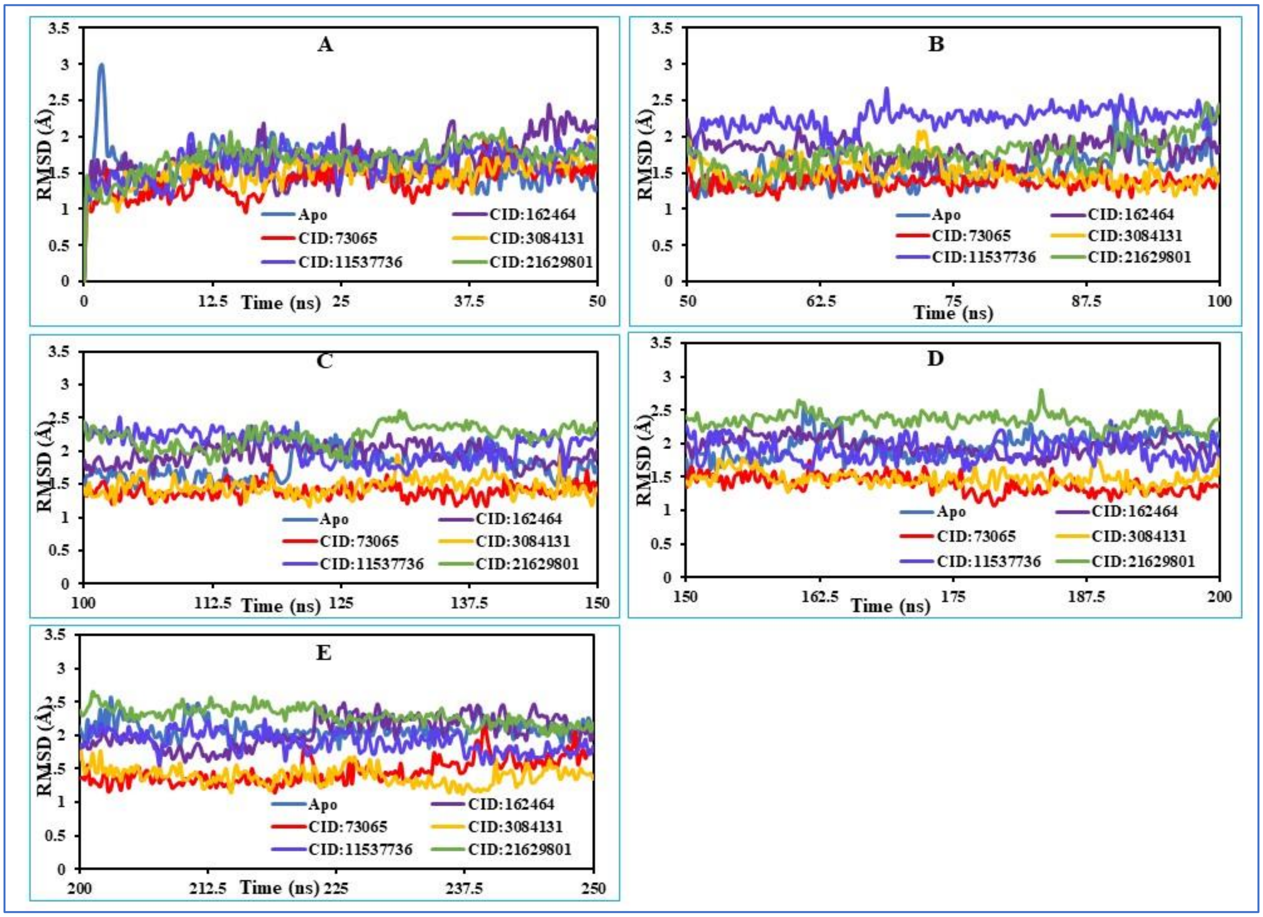


**Figure S6:** Representing multiple repetition of RMSD value for each 50 ns interval obtained from 250 ns Simulation time. Herein, A. 1-50 ns, B. 50-100 ns, C. 100-150 ns, D. 150-200 ns, and E. 200-250 ns RMSD of the selected protein ligand's complex structure.

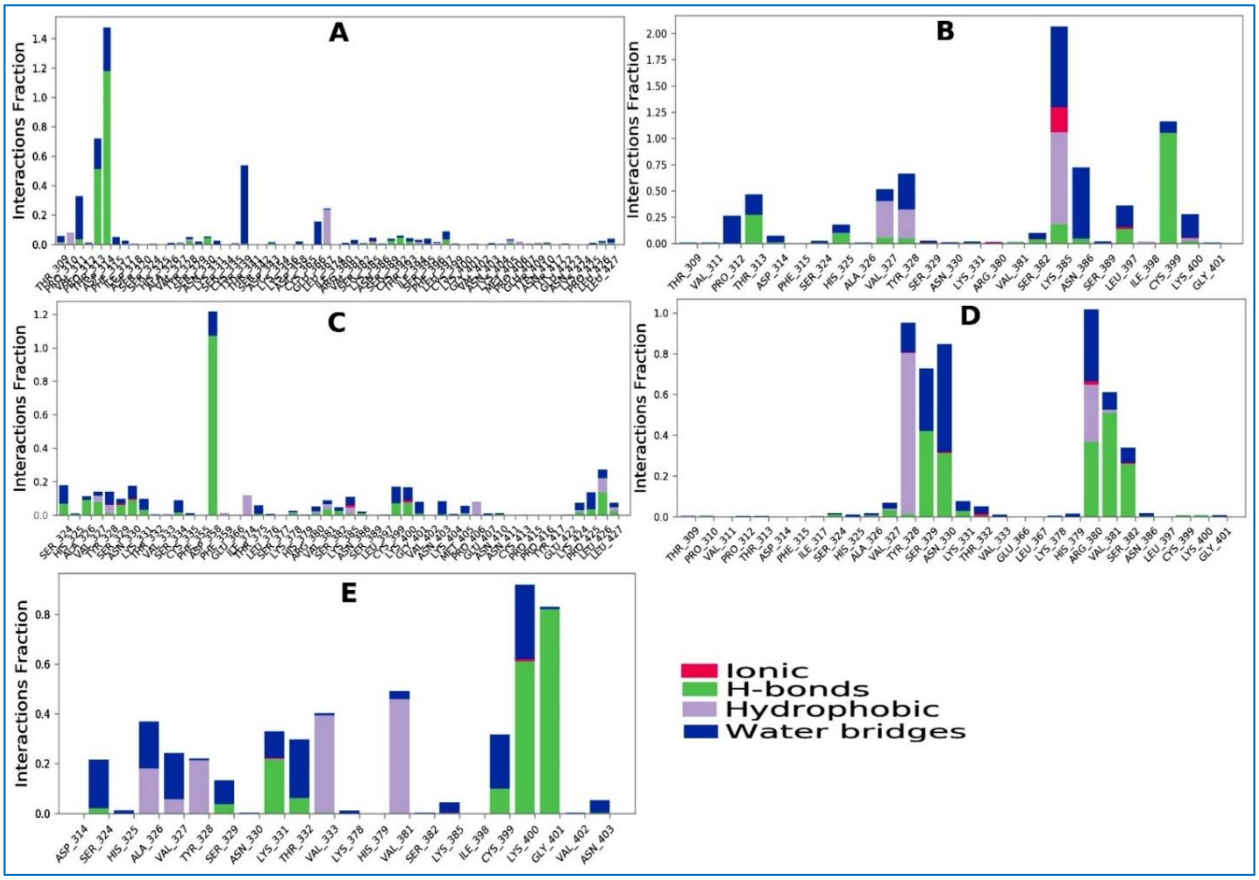


**Figure S7.** The stacked bar charts representing the protein-ligands interactions found during the 250 ns simulation run. Herein, (A). PubChem CID:73065, (B). PubChem CID:3084131, (C). PubChem CID:11537736, (D). PubChem CID:21629801, and (E). PubChem CID:162464 compounds in complex with the MCPyV LT protein.
