# Supplementary material for: Toward the Identification of Natural Antiviral Drug Candidates against Merkel Cell Polyomavirus: Computational Drug Design Approaches
Source: Pharmaceuticals (Basel). 2022 Apr 20;15(5):501. doi: 10.3390/ph15050501 (PMC9146542; doi:10.3390/ph15050501)
Supplement: Supplementary file 1 [file pharmaceuticals-15-00501-s001.zip › Supplimentary Table (F).pdf]

Supplementary Table

Towards the Identification of Natural Bioactive Antiviral Drug Candidates Against Merkel Cell Polyomavirus: Computational Drug Design Approaches

Table S1. List of plant and their antiviral activity against different virus, which compounds has been retrieved from IMPPAT database for this study.

Plant Name	Activity Against	Reference
Abies pindrow	Influenza	[67][68]
Acacia nilotica	Hepatitis C virus (HCV)	[4][8]
Actaea racemosa	Zika Virus	[78][79]
Aegle marmelos	Human coxsackieviruses B1-B6	[71][73][80]
Aglaia odorata	Herpes simplex virus type 1	[1][9]
Agrimonia pilosa	HSV -1	[69][70]
Ailanthus altissima	HIV	[69]
Aizoon canariense	Herpes Simplex Virus,Polio virus	[42][43][44]
Allium cepa	Herpes simplex virus type 1	[88]
Allium sativum	Influenza virus H9N2	[69][80][85]
Alpinia galanga	Epstein-Barr virus	[1][10][11][12]
Alpinia officinarum	Respiratory syncytial virus, poliovirus, measles virus, and herpes simplex virus type 1	[89]
Andrographis paniculata	HIV	[6][13][14]
Apium graveolens	Alfalfa mosaic virus (AMV)	[45]
Artemis Annua	Bovine viral diarrhoea virus (BVDV), HSV-1	[51]
Artemisia absinthium	HIV	[57][58]
Artemisia capillaris	HBV	[103]
Artocarpus lacucha	HSV-1 HSV-2	[5][15]
Asparagus racemosus	SARS-CoV-2	[105]
Atalantia monophylla	HSV	[104]
Azadirachta indica	Dengue Virus Type 2	[107]
Bauhinia variegata	HSV -1, HSV -2, Vaccinia virus	[73][80][82]

Berberis vulgaris	HIV-1	[80][83]
Boehmeria nivea	HBV	[107]
Boerhavia diffusa	Viral Hepatitis	[108]
Caesalpinia sappan	HIV	[4][16]
Calotropis procera	Canine parainfluenza virus-2, canine distemper virus, Infectious Bovine Rhinotracheitis (IBR) virus, lumpy skin disease virus, feline herpesvirus-1 (FHV-1)	[106]
Camellia sinensis	Herpes simplex virus, Adenovirus, Influenza A and B virus, Bovine coronavirus and Bovine rotavirus, Epstein-barr virus, Human immunodeficiency virus type 1, HBV	[70][71][72][73][74]
Capparis spinosa	HIV-1	[109][110]
Carum Carvi	HSV-1	[46][47][48]
Cassia occidentalis	HIV	[102]
Cassine xylocarpa	HIV	[111]
Castanospermum australe	HIV	[104]
Curcuma aromatica	HCV	[106]
Curcuma longa	HSV	[112][113]
Daucus carota	HSV-1	[70][71]
Dianthus caryophyllus	HSV-1, HAV	[64][65][66]
Diospyros kaki	Influenza, HSV	[114][115]
Dittrichia viscosa	HIV	[49]
Eleusine indica	HSV	[116]
Euphorbia hirta	HIV-1, HIV-2, SIV mac 251	[67][71]
Fagonia cretica	SARS-COV-2	[100][101]
Fagopyrum esculentum	Vesicular stomatitis virus	[80]
Ficus benjamina	HSV-1,2	[117][118]
Ginkgo biloba	Influenza virus, Human alphaherpesvirus 1 (HHV-1), Human alphaherpesvirus 2 (HHV-2)	[75][80][84]
Glycyrrhiza glabra	HEP-A,B,C ' HIV,SARS-COV,EV71	[52][53]
Gmelina arborea	enterovirus (type 71, BrCr)	[90]
Grewia asiatica	Urdbean Leaf Crinkle Virus (ULCV)	[59]

Guazuma ulmifolia	Polio Virus	[107]
Hemidesmus indicus	HIV	[4][17][18]
Holoptelea integrifolia	Herpes simplex virus	[1][20][21][22]
Hyssopus officinalis	HIV	[102]
Justicia gendarussa	HIV-1	[106]
Kalanchoe pinnata	Chikungunya virus	[2][23][24][25][26][27][28]
Lawsonia inermis	Sembiki forest virus (SFV), Epstein-Barr virus	[62][63]
Lilium candidum	HSV-1, HSV-2	[3][29][30]
Lycoris radiata	SARS-COV	[107][119][120]
Magnolia officinalis	Dengue-Virus Type 2	[121][122]
Melastoma malabathricum	Polio Virus	[106]
Melia azedarach	VSV, HSV-1	[5]
Melissa officinalis	HSV	[6]
Mentha arvensis	HSV-1	[102]
Mentha piperita	NDV, West Nile Virus	[91][99]
Micromeria capitellata	HSV-1	[91][97][98]
Mimosa pudica	Mumps Virus	[123]
Mirabilis jalapa	TMV	[41]
Morinda citrifolia	HIV-1	[106]
Moringa oleifera	HIV, HSV, EBV etc	[7]
Nigella sativa	Multiple viruses, MCMV infection, Viral strain MHV-A59	[69][72][73][74]
Ocimum basilicum	Adenoviruses and Enteroviruses	[1][31]
Panax Ginseng	EV71, HHV, HIV	[54][55][56]
Papaver somniferum	HSV	[104]
Petiveria alliacea	Bovine viral diarrhea virus	[124]
Petroselinum crispum	moloney murine leukemia virus (PIM-1)	[87]
Phyllanthus acidus	HBV	[71]
Phyllanthus amarus	HIV	[107][125]
Phyllanthus emblica	HIV	[102]
Phyllanthus reticulatus	HIV	[96]
Phyllanthus urinaria	HSV	[70][72][75]
Piper longum	Dengue	[39][40]

Plumbago zeylanica	Coxsackie virus B3	[106]
Prunella vulgaris	HIV-1, Ebola virus	[3][34]
Punica granatum	Herpes simplex virus type 1	[1][32][33]
Rheum officinale	HSV -1	[70][72][76]
Rheum palmatum	RSV, CVB-5	[50]
Salix babylonica	Ranikhet disease virus (RDV)	[95]
Sambucus nigra	Viral Replication	[102]
Schinus molle	HSV-1	[93][94]
Scutellaria baicalensis	(H3N2) and B Viruses	[92]
Scutellaria baicalensis	Influenza B virus, Influenza A (H3N2) virus	[106]
Silybum marianum	CHIKV, influenza A virus, HCV	[78]
Solanum nigrum	Tobacco mosaic virus, sunnhemp rosette virus	[80]
Spondias mombin	HSV	[126][127]
Stephania japonica	HSV	[104]
Swertia chirata	HSV-1	[102]
Syzygium aromaticum	Herpes Simplex, Hepacitis C	[60][61]
Tanacetum vulgare	HSV-1, HSV-2	[5][35][36]
Terminalia chebula	Human Cytomegalovirus, HCV, Dengue Virus, Measles	[106]
Thymus serpyllum	Newcastle disease (NDV)	[91]
Tuberaria lignosa	HIV	[128]
Vitis vinifera	Influenza A, HSV and varicella-zoster	[73][77]
Zataria multiflora	HSV-1	[3][37][38
Zingiber officinale	NDV	[106]
Ziziphus mauritiana	Dengue Virus	[73][80][86]

Table S2. Active pocket of the MCPYV LT protein (PDB ID: 3QFQ; Chain: A) with surface area and volume.

Pocket ID	Area (SA)	Volume (SA)
1	103.341	37.747
2	39.623	17.969
3	28.757	3.447

4	4.777	1.084
5	5.215	0.394
6	1.642	0.260
7	2.339	0.135
8	0.680	0.041
9	0.933	0.040
10	1.185	0.037
11	0.615	0.017
12	0.131	0.002
13	0.149	0.002
14	0.068	0.001
15	0.097	0.001
16	0.031	0.000
17	0.064	0.000
18	0.052	0.000
19	0.001	0.000

Table S3. First four active pocket and there corresponding AA residues retrieved from the MCPYV LT protein (PDB ID: 3QFQ; Chain: A)

Pocket ID	Chain	Sequence ID	AA
1	A	324	SER
		326	ALA
		327	VAL
		329	SER
		331	LYS
		333	VAL
		380	ARG
		381	VAL
		382	SER
2	A	358	ASP
		359	PHE
		374	ILE
		405	MET
		406	PRO
		426	LEU
		427	LEU
3	A	340	THR

		344	LYS
		347	GLU
		348	LEU
		388	CYS
		391	PHE
		392	CYS
		394	ILE
		395	SER
		396	PHE
		397	LEU
4	A	325	HIS
		326	ALA
		327	VAL
		400	LYS

Table S4. A comparative docking score of the compound that has found during the docking between the protein with water and without water.

Ligand	Binding Affinity without water (kcal/mol)	Binding Affinity with water (kcal/mol)
CID:3084131	-7.6	-7.3
CID:11537736	-6.7	-6.1
CID:21629801	-7.1	-6.6
CID:162464	-6.5	-6.4
CID:73065	-6.6	-5.8

Table S5. List of bonding interactions between selected five phytochemical with MCPyV large T antigen.

ID		Residues	Distance (Å)	Bond category	Bond type
CID:73065		LYS385	5.06	Hydrophobic	Pi- Alkyl
		VAL381	5.45	Hydrophobic	Pi- Alkyl
		VAL381	3.46	Hydrophobic	Pi- Sigma
		VAL381	3.44	Hydrophobic	Pi- Sigma
		LYS331	2.66	N/A	Unfavorable
		LYS331	3.69	Hydrophobic	Alkyl
		VAL333	4.85	Hydrophobic	Alkyl
		SER324	1.89	Hydrogen	Conventional Hydrogen
		SER324	2.97	Hydrogen	Carbon Hydrogen

		ALA326	3.91	Hydrophobic	Alkyl
		ALA326	2.43	Hydrogen	Conventional Hydrogen
		ALA326	4.23	Hydrophobic	Pi- Alkyl
		ALA326	5.37	Hydrophobic	Pi- Alkyl
CID:11537736		ALA326	4.93	Hydrophobic	Pi- Alkyl
		ARG380	5.16	Hydrophobic	Pi- Alkyl
		VAL381	3.76	Hydrophobic	Pi- Sigma
		VAL381	4.97	Hydrophobic	Pi- Alkyl
		SER382	3.57	Hydrophobic	Pi- Sigma
		LYS385	4.54	Hydrophobic	Pi- Alkyl
CID:21629801		ARG380	2.35	Hydrogen	Conventional Hydrogen
		VAL381	2.95	Hydrogen	Conventional Hydrogen
		SER382	2.07	Hydrogen	Conventional Hydrogen
		ASN330	2.05	Hydrogen	Conventional Hydrogen
		CYS399	2.84	Hydrogen	Conventional Hydrogen
		SER382	3.21	Hydrogen	Carbon Hydrogen
		SER382	3.21	Hydrogen	Carbon Hydrogen
		SER324	2.94	Hydrogen	Pi-Donor Hydrogen
		LYS331	3.61	Hydrophobic	Pi-Sigma
		VAL381	5.31	Hydrophobic	Alkyl
		LYS385	4.48	Hydrophobic	Pi-Alkyl
		ALA326	4.06	Hydrophobic	Pi-Alkyl
		VAL333	4.62	Hydrophobic	Pi-Alkyl
		VAL381	5.41	Hydrophobic	Pi-Alkyl
CID:162464		LYS385	3.72	Hydrophobic	Alkyl
		LYS385	4.72	Hydrophobic	Pi- Alkyl
		VAL381	4.08	Hydrophobic	Alkyl
		VAL381	5.28	Hydrophobic	Pi- Alkyl
		LYS331	3.37	Hydrogen	Carbon Hydrogen
		SER329	1.95	Hydrogen	Conventional Hydrogen

		LYS400	2.74	Hydrogen	Conventional Hydrogen
		CYS399	2.47	Hydrogen	Conventional Hydrogen
		LEU397	3.55	Hydrogen	Carbon Hydrogen
CID:3084131		SER329	2.15	Hydrogen	Conventional hydrogen
		SER324	2.97	Hydrogen	Conventional hydrogen
		VAL 381	3.81	Hydrogen	Pi-Donor Hydrogen
		VAL 381	3.52	Hydrophobic	Pi-Sigma
		ALA 326	4.93	Hydrophobic	Pi-Alkyl
		ARG380	5.17	Hydrophobic	Pi-Alkyl

Table S6. The contributions to the total energy from different components analysis through MM-GBSA energy calculation method. Analysis of MM-GBSA for selected five compounds found a significant contribution in the binding, coulomb, covalent, van der waals, lipophilic, and generalized born electrostatic solvation energy.

Complex Name	ΔG Bind	ΔG Bind Coulomb	ΔG Bind Covalent	ΔG Bind Hbond	ΔG Bind Lipo	ΔG Bind Packing	ΔG Bind Solv GB	ΔG Bind vdW
CID:162464	-47.91 ±3.92	-12.969 ±4.37	1.76 ±1.56	-1.19 ±0.53	-8.49 ±1.39	-0.22 ±0.37	16.85 ±2.69	-43.67 ±3.11
CID: 73065	-29.69 ±9.53	-14.75 ±6.36	0.80 ±0.80	-1.70 ±0.81	-8.488 ±2.52	-0.04 ±0.21	13.82 ±4.66	-19.33 ±5.44
CID:3084131	-39.98 ±7.19	-49.33 ±16.01	2.58 ±.407	-2.08 ±0.64	-9.70 ±1.68	-0.10 ±0.18	48.931 ± 14.07	-30.26 ±3.78
CID:11537736	-28.34 ±10.04	-16.85 ±7.22	1.10 ±1.61	-1.87 ± 0.76	-7.56 ±2.72	-0.43 ±0.65	17.028 ± 4.09	-19.75 ±5.77
CID:21629801	-36.66 ±11.0	-20.86 ±7.58	2.20 ±1.52	-1.82 ± 0.76	-5.16 ±1.94	-3.79 ±2.08	19.87 ±5.68	-27.08 ± 7.16

References

[1] Hafidh, R. R., et al. "Asia is the mine of natural antiviral products for public health." The Open Complementary Medicine Journal 1.1 (2009).

[2] Joshi, Bishnu, et al. "Antibacterial, antifungal, antiviral, and anthelmintic activities of medicinal plants of Nepal selected based on ethnobotanical evidence." Evidence-Based Complementary and Alternative Medicine 2020 (2020).

[3] Ben-Shabat, Shimon, et al. "Antiviral effect of phytochemicals from medicinal plants: applications and drug delivery strategies." Drug Delivery and Translational Research 10.2 (2020): 354-367.

[4] Mohan, Syam, et al. "Bioactive natural antivirals: An updated review of the available plants and isolated molecules." Molecules 25.21 (2020): 4878.

[5] Kapoor, R., B. Sharma, and S. S. Kanwar. "Antiviral phytochemicals: an overview." Biochem Physiol 6.2 (2017): 7.

[6] Naithani, Rajesh, et al. "Antiviral activity of phytochemicals: a comprehensive review." Mini reviews in medicinal chemistry 8.11 (2008): 1106-1133.

[7] Biswas, D., et al. "Moringa oleifera Lam. and derived phytochemicals as promising antiviral agents: A review." South African Journal of Botany 129 (2020): 272-282.

[8] Shahzan, M. Sohaib, AS Smiline Girija, and J. Vijayashree Priyadharsini. "A computational study targeting the mutated L321F of ERG11 gene in C. albicans, associated with fluconazole resistance with bioactive compounds from Acacia nilotica." Journal de mycologie medicale 29.4 (2019): 303-309.

[9] Efdi, Mai, et al. "Chemical constituents of Aglaia odorata leaves and their anti-inflammatory effects." Natural Product Communications 12.11 (2017): 1717-1720.

[10] Ghosh, S., Rangan, L. Alpinia: the gold mine of future therapeutics. 3 Biotech 3, 173–185 (2013). https://doi.org/10.1007/s13205-012-0089-x

[11] Herrmann, Florian, et al. "Diversity of pharmacological properties in Chinese and European medicinal plants: cytotoxicity, antiviral and antitrypanosomal screening of 82 herbal drugs." Diversity 3.4 (2011): 547-580.

[12] Chudiwal, A. K., D. P. Jain, and R. S. Somani. "Alpinia galanga Willd.–An overview on phyto-pharmacological properties." (2010).

[13] Kalaivani, C. S., et al. "GC-MS studies on Andrographis paniculata (Burm. f.) Wall. Ex Nees—a medicinally important plant." Int J Med Arom Plants 2.1 (2012): 69-74.

[14] Rajagopal, Kalirajan, et al. "Activity of phytochemical constituents of Curcuma longa (turmeric) and Andrographis paniculata against coronavirus (COVID-19): an in silico approach." Future journal of pharmaceutical sciences 6.1 (2020): 1-10.

[15] Noikotra, Kowit, et al. "Phytochemicals, cytotoxicity, and genotoxicity of three Artocarpus species reveal arbutin in A. lacucha." Sci Asia 44 (2018): 170-8.

[16] Pawar, Chaitali R., Amol D. Landge, and Sanjay J. Surana. "Phytochemical and pharmacological aspects of Caesalpinia sappan." Journal of Pharmacy Research 1.2 (2008): 131-138.

[17] Swathi, S., P. Amareshwari, and A. Venkatesh Kand Roja Rani. "Phytochemical and pharmacological benefits of Hemidesmus indicus: An updated review." Journal of Pharmacognosy and Phytochemistry 8.1 (2019): 256-262.

[18] Banerjee, Aparna, and Subha Ganguly. "Medicinal importance of Hemidesmus indicus: a review on its utilities from ancient Ayurveda to 20th Century." Adv Biores 5.3 (2014): 208-13.

[19] Das, Sarita, and Satpal Singh Bisht. "The bioactive and therapeutic potential of Hemidesmus indicus R. Br.(Indian Sarsaparilla) root." Phytotherapy Research 27.6 (2013): 791-801.
[20] Sandhar, Harleen Kaur, et al. "Chemistry and medicinal properties of Holoptelea integrifolia." International journal of drug development and research 3.1 (2011): 6-11.
[21] Mondal, S. U. M. I. T. A., and A. Bandyopadhyaya. "The wonders of a medicinal tree: Holoptelea Integrifolia (ROXB.) Planch." International Journal of Pharmacy and Pharmaceutical Sciences 8.8 (2016): 43-48.
[22] Kumar, Vijay, et al. "Phytochemical, analytical and medicinal studies of holoptelea integrifolia roxb. Planch-a review." Current Traditional Medicine 5.4 (2019): 270-277.
[23] Quazi Majaz, A., et al. "The miracle plant (Kalanchoe pinnata): a phytochemical and pharmacological review." Int J Res Ayurveda Pharm 2.5 (2011): 1478-82.
[24] Pattewar, Seema V. "Kalanchoe pinnata: phytochemical and pharmacological profile." International Journal of Pharmaceutical Sciences and Research 3.4 (2012): 993.
[25] Majaz, Quazi, et al. "Phytochemical analysis of chloroform extract of roots of Kalanchoe pinnata by HPLC and GCMS." Int J Pharm Sci Res 2 (2011): 1693-9.
[26] Supratman, Unang, et al. "New insecticidal bufadienolide, bryophyllin C, from Kalanchoe pinnata." Bioscience, biotechnology, and biochemistry 64.6 (2000): 1310-1312.
[27] El Abdellaoui, Saïda, et al. "Bioactive molecules in Kalanchoe pinnata leaves: extraction, purification, and identification." Analytical and bioanalytical chemistry 398.3 (2010): 1329-1338.
[28] George, Latha Ophelia, H. R. Radha, and B. V. Somasekariah. "In vitro anti-diabetic activity and GC-MS analysis of bioactive compounds present in the methanol extract of Kalanchoe pinnata." (2018).
[29] Zaccai, Michele, et al. "Medicinal Properties of Lilium candidum L. and Its Phytochemicals." Plants 9.8 (2020): 959.
[30] Yarmolinsky, Ludmila, et al. "Antiviral activity of ethanol extracts of Ficus binjamina and Lilium candidum in vitro." New biotechnology 26.6 (2009): 307-313.
[31] Khair-ul-Bariyah, S., D. Ahmed, and M. Ikram. "Ocimum basilicum: a review on phytochemical and pharmacological studies." Pak. J. Chem 2.2 (2012): 78-85.
[32] Jasuja, Nakuleshwar Dut, et al. "Pharmacological characterization and beneficial uses of Punica granatum." Asian Journal of Plant Sciences 11.6 (2012): 251.
[33] Singh, Balwinder, et al. "Phenolic compounds as beneficial phytochemicals in pomegranate (Punica granatum L.) peel: A review." Food chemistry 261 (2018): 75-86.
[34] Chen, Yuhang, et al. "Variation in concentrations of major bioactive compounds in Prunella vulgaris L. related to plant parts and phenological stages." Biological Research 45.2 (2012): 171-175.
[35] Ivănescu, B. I. A. N. C. A., et al. "Antioxidant, antimicrobial and cytotoxic activity of Tanacetum vulgare, Tanacetum corymbosum and Tanacetum macrophyllum extracts." Farmacia 66.2 (2018): 282-288.
[36] Mureşan, Maria Lucia, et al. "Botanical and phytochemical studies on Tanacetum vulgare L. from Transylvania." Acta Medica Transilvanica 2.4 (2014): 145-147.
[37] Sajed, Hassan, Amirhossein Sahebkar, and Mehrdad Iranshahi. "Zataria multiflora Boiss.(Shirazi thyme)—an ancient condiment with modern pharmaceutical uses." Journal of ethnopharmacology 145.3 (2013): 686-698.
[38] Saedi Dezaki, Ebrahim, et al. "Chemical composition along with anti-leishmanial and cytotoxic activity of Zataria multiflora." Pharmaceutical biology 54.5 (2016): 752-758.

[39] Zaveri, M., Khandhar, A., Patel, S., & Patel, A. (2010). Review Article CHEMISTRY AND PHARMACOLOGY OF PIPER LONGUM L. International Journal of Pharmaceutical Sciences Review and Research, 5(1), 234–238.

[40] Kumar, S., Kamboj, J., Suman, & Sharma, S. (2011). Overview for Various Aspects of the Health Benefits of Piper Longum Linn. Fruit. JAMS Journal of Acupuncture and Meridian Studies, 4(2), 134–140. https://doi.org/10.1016/S2005-2901(11)60020-4

[41] Hanani, E., Prastiwi, R., & Karlina, L. (2017). Indonesian mirabilis jalapa linn.: A pharmacognostical and preliminary phytochemical investigations. Pharmacognosy Journal, 9(5), 683–688. https://doi.org/10.5530/pj.2017.5.108

[42] El-Amier, Y. A., & Al-Hadithy, O. N. (2020). Phytochemical constituents, antioxidant and allelopathic activities of Aizoon canariense L. On Zea mays (L.) and associated weeds. Plant Archives, 20(1), 303–310.

[43] El-amier, Y. A., Haroun, S. A., El-shehaby, O. A., & Al-hadithy, O. N. (2016). Antioxidant and Antimicrobial Properties of Some Wild Aizoaceae Species Antioxidant and Antimicrobial Properties of Some Wild Aizoaceae. Journal of Environmental Sciences ·, 45(1), 1–10.

[44] Noor, A., Gunasekaran, S., & Vijayalakshmi, M. A. (2018). Article in Pharmacognosy Research · October 2017. Pharmacognosy Research, 10(October), 24–30. https://doi.org/10.4103/pr.pr

[45] Bello, O. A., Ayanda, O. I., Aworunse, O. S., & Olukanmi, B. I. (2018). Pharmacognosy Reviews, 1(2), 8–15. https://doi.org/10.4103/phrev.phrev

[46] Sharma, A., Sharma, P., Singh Tuli, H., & Sharma, A. K. (2018). Phytochemical and Pharmacological Properties of Flavonols. ELS, (June), 1–12. https://doi.org/10.1002/9780470015902.a0027666

[47] Ali Esmail Al-Snafi. (2015). Indian Journal of Pharmaceutical Science & Research ADHERENCE – A REVIEW. Ijpsrjournal, 5(2), 72–82.

[48] Joshi, R. K., & Soulimani, R. (2020). Ethno-medicinal and phytochemical potential of Carum carvi Linn. and Cuminum cyminum: A review. International Journal of Pharmacognosy and Life Science, 1(1), 33–37. https://doi.org/10.33545/27072827.2020.v1.i1a.7

[49] Grauso, L., Cesarano, G., Zotti, M., Ranesi, M., Sun, W., Bonanomi, G., & Lanzotti, V. (2020). Exploring Dittrichia viscosa (L.) Greuter phytochemical diversity to explain its antimicrobial, nematicidal and insecticidal activity. Phytochemistry Reviews, 19(3), 659–689. https://doi.org/10.1007/s11101-019-09607-1

[50] KHATTAK, A. K., HASSAN, S. M., & MUGHAL, S. S. (2020). General Overview of Phytochemistry and Pharmacological Potential of Rheum Palmatum (Chinese Rhubarb). Innovare Journal of Ayurvedic Sciences, 8(6), 5–9. https://doi.org/10.22159/ijas.2020.v8i6.39192

[51] Czechowski, T., Larson, T. R., Catania, T. M., Harvey, D., Wei, C., Essome, M., … Graham, I. A. (2018). Detailed phytochemical analysis of high- and low artemisinin-producing chemotypes of artemisia annua. Frontiers in Plant Science, 9(May), 1–14. https://doi.org/10.3389/fpls.2018.00641

[52] Mamedov, N. A., & Egamberdieva, D. (2019). Phytochemical constituents and pharmacological effects of licorice: A review. Plant and Human Health: Pharmacology and Therapeutic Uses, 3, 1–21. https://doi.org/10.1007/978-3-030-04408-4_1

[53] Al-snafi, A. E. (2018). Glycyrrhiza glabra : A phytochemical and pharmacological review Glycyrrhiza glabra : A phytochemical and pharmacological review Prof Dr Ali Esmail Al-Snafi. IOSR Journal of Pharmacy, 8(6), 1–17.

[54] Im, K., Kim, J., & Min, H. (2016). Ginseng, the natural effectual antiviral: Protective effects of Korean red ginseng against viral infection. Journal of Ginseng Research, 40(4), 309–314. https://doi.org/10.1016/j.jgr.2015.09.002

[55] Ru, W., Wang, D., Xu, Y., He, X., Sun, Y. E., Qian, L., … Qin, Y. (2011). Chemical constituents and bioactivities of Panax ginseng (C. A. Mey.). Drug Discoveries & Therapeutics, 9(1), 23–32. https://doi.org/10.5582/ddt.2015.01004

[56] Tripathi, R. D., & Tiwari, K. P. (1981). Phytochemical investigation of the roots of Tacca aspera. Planta Medica, 41(4), 414–415. https://doi.org/10.1055/s-2007-971741

[57] Msaada, K., Salem, N., Bachrouch, O., Bousselmi, S., Tammar, S., Alfaify, A., … Marzouk, B. (2015). Chemical composition and antioxidant and antimicrobial activities of wormwood (Artemisia absinthium L.) essential oils and phenolics. Journal of Chemistry, 2015. https://doi.org/10.1155/2015/804658

[58] Article, R. (2010). Available Online through HEPATOPROTECTIVE ACTIVITY – A REVIEW, 2(3), 354–366.

[59] Pundlik, S. P. (2020). Pharmacognostic Study On Leaves Of Genus Grewia From Western Maharashtra, 29(6), 8797–8802.

[60] Mittal, M., Gupta, N., Parashar, P., Mehra, V., & Khatri, M. (2014). Phytochemical evaluation and pharmacological activity of syzygium aromaticum: A comprehensive review. International Journal of Pharmacy and Pharmaceutical Sciences, 6(8), 67–72.

[61] Kaur, K., & Kaushal, S. (2019). Phytochemistry and pharmacological aspects of Syzygium aromaticum : A review. Journal of Pharmacognosy and Phytochemistry, 8(1), 398–406.

[62] Divaricata, E., & Racemosus, A. (2010). Thenmoxhi M, (9), 86–91.

[63] Gull, I., Sohail, M., Aslam, M. S., & Athar, M. A. (2013). Phytochemical, toxicological and antimicrobial evaluation of lawsonia inermis extracts against clinical isolates of pathogenic bacteria. Annals of Clinical Microbiology and Antimicrobials, 12(1), 1–6. https://doi.org/10.1186/1476-0711-12-36

[64] Galeotti, F., Barile, E., Curir, P., Dolci, M., & Lanzotti, V. (2008). Flavonoids from carnation (Dianthus caryophyllus) and their antifungal activity. Phytochemistry Letters, 1(1), 44–48. https://doi.org/10.1016/j.phytol.2007.10.001

[65] Al-Snafi, P. D. A. E. (2017). Chemical contents and medical importance of Dianthus caryophyllus- A review. IOSR Journal of Pharmacy (IOSRPHR), 07(03), 61–71. https://doi.org/10.9790/3013-0703016171

[66] Chandra, S., Rawat, D. S., Chandra, D., & Rastogi, J. (2016). Nativity, phytochemistry,ethnobotany and pharmacology of Dianthus caryophyllus. Research Journal of Medicinal Plant, 10(1), 1–9. https://doi.org/10.3923/rjmp.2016.1.9

[67] R. Amber, M. Adnan, A. Tariq, and S. Mussarat, "A review on antiviral activity of the Himalayan medicinal plants traditionally used to treat bronchitis and related symptoms," J. Pharm. Pharmacol., vol. 69, no. 2, pp. 109–122, 2017, doi: 10.1111/jphp.12669.

[68] M. Rajbhandari et al., "Antiviral Activity of Some Plants Used in Nepalese Traditional Medicine," Evidence-Based Complement. Altern. Med., vol. 6, no. 4, pp. 517–522, 2009, doi: 10.1093/ecam/nem156.

[69] M. Babar, S. Z. Najam-us-Sahar, M. Ashraf, and A. G. Kazi, "Antiviral drug therapy- exploiting medicinal plants," J. Antivirals Antiretrovir., vol. 5, no. 2, pp. 28–36, 2013, doi: 10.4172/jaa.1000060.

[70] P. Ruwali, N. Rai, N. Kumar, and P. Gautam, "Antiviral Potential of Medicinal Plants: an Overview," Int. Res. J. Pharm., vol. 4, no. 6, pp. 8–16, 2013, doi: 10.7897/2230-8407.04603.

[71] S. Ben-Shabat, L. Yarmolinsky, D. Porat, and A. Dahan, “Antiviral effect of phytochemicals from medicinal plants: Applications and drug delivery strategies,” Drug Deliv. Transl. Res., vol. 10, no. 2, pp. 354–367, 2020, doi: 10.1007/s13346-019-00691-6.

[72] S. A. Jassim and M. A. Naji, “Novel antiviral agents: A medicinal plant perspective,” J. Appl. Microbiol., vol. 95, no. 3, pp. 412–427, 2003, doi: 10.1046/j.1365-2672.2003.02026.x.

[73] I. Fatima, S. Kanwal, and T. Mahmood, “Natural Products Mediated Targeting of Virally Infected Cancer,” Dose-Response, vol. 17, no. 1, pp. 1–16, 2019, doi: 10.1177/1559325818813227.

[74] J. S. Mani et al., “Natural product-derived phytochemicals as potential agents against coronaviruses: A review,” Virus Res., vol. 284, no. June, 2020, doi: 10.1016/j.virusres.2020.197989.

[75] M. Mukhtar, M. Arshad, M. Ahmad, R. J. Pomerantz, B. Wigdahl, and Z. Parveen, “Antiviral potentials of medicinal plants,” Virus Res., vol. 131, no. 2, pp. 111–120, 2008, doi: 10.1016/j.virusres.2007.09.008.

[76] C. Debprasad and S. Das, “Herbal Medicinal Products against Herpesvirus diseases Ethnomedicines in Antiviral Drug Discovery,” no. March 2019, 2009.

[77] V. Berardi, F. Ricci, M. Castelli, G. Galati, and G. Risuleo, “Resveratrol as an antiviral against polyomavirus,” Recent Adv. Microbiol., no. May 2016, pp. 30–37, 2011, doi: 10.1201/b12222-4.

[78] H. Zakaryan, E. Arabyan, A. Oo, and K. Zandi, “Flavonoids: promising natural compounds against viral infections,” Arch. Virol., vol. 162, no. 9, pp. 2539–2551, 2017, doi: 10.1007/s00705-017-3417-y.

[79] K. G. Byler, I. V. Ogungbe, and W. N. Setzer, “In-silico screening for anti-Zika virus phytochemicals,” J. Mol. Graph. Model., vol. 69, no. January, pp. 78–91, 2016, doi: 10.1016/j.jmgm.2016.08.011.

[80] R. M. Perez G., “Antiviral activity of compounds isolated from plants,” Pharm. Biol., vol. 41, no. 2, pp. 107–157, 2003, doi: 10.1076/phbi.41.2.107.14240.

[81] Z. A. Torky, “Antiviral Activity of Polyphenols Extracts From Daucus carota against Herpes Simplex Virus type 1,” Tojsat, vol. 3, no. 1, pp. 20–32, 2013.

[82] K. A. Parmar, Prajapati, and S. N., “HPTLC-aided phytochemical fingerprint analysis as a tool for evaluation and antiviral activity using HeLa cell cultures of Bauhinia variegata plant.,” Asian Journal of Experimental Chemistry, 2009. https://www.cabdirect.org/cabdirect/abstract/20103195343 (accessed Jun. 15, 2021).

[83] A. Warowicka, R. Nawrot, and A. Goździcka-Józefiak, “Antiviral activity of berberine,” Arch. Virol., vol. 165, no. 9, pp. 1935–1945, 2020, doi: 10.1007/s00705-020-04706-3.

[84] M. Sochocka, M. Sobczyński, M. Ochnik, K. Zwolińska, and J. Leszek, “Hampering Herpesviruses HHV-1 and HHV-2 Infection by Extract of Ginkgo biloba (EGb) and Its Phytochemical Constituents,” Front. Microbiol., vol. 10, p. 2367, Oct. 2019, doi: 10.3389/fmicb.2019.02367.

[85] A. Rasool et al., “Anti-Avian influenza virus H9N2 activity of aqueous extracts of Zingiber officinalis (Ginger) & Allium sativum (Garlic) in chick embryos,” Pak. J. Pharm. Sci., vol. 30, no. 4, pp. 1341–1344, 2017.

[86] R. Batool, E. Aziz, T. Mahmood, B. Tan, and V. Chow, “Inhibitory activities of extracts of Rumex dentatus, Commelina benghalensis, Ajuga bracteosa, Ziziphus mauritiana as well as their compounds of gallic acid and emodin against dengue virus,” Asian Pac. J. Trop. Med., vol. 11, no. 4, pp. 265–271, Apr. 2018, doi: 10.4103/1995-7645.231466.

[87] A. N. Bullock, J. É. Debreczeni, O. Y. Fedorov, A. Nelson, B. D. Marsden, and S. Knapp, “Structural basis of inhibitor specificity of the human protooncogene proviral insertion site in moloney murine leukemia virus (PIM-1) kinase,” J. Med. Chem., 2005, doi: 10.1021/jm0504858.

[88] R. M. Romeilah, S. A. Fayed, and G. I. Mahmoud, “Chemical compositions, antiviral and antioxidant activities of seven essential oils,” J. Appl. Sci. Res., 2010.

[89] K. Konno et al., “Antiviral activities of diarylheptanoids isolated from Alpinia officinarum against respiratory syncytial virus, poliovirus, measles virus, and herpes simplex virus type 1 in vitro,” Nat. Prod. Commun., 2011, doi: 10.1177/1934578x1100601222.

[90] S. K. Panda, L. Padhi, P. Leyssen, M. Liu, J. Neyts, and W. Luyten, “Antimicrobial, anthelmintic, and antiviral activity of plants traditionally used for treating infectious disease in the Similipal Biosphere Reserve, Odisha, India,” Front. Pharmacol., 2017, doi: 10.3389/fphar.2017.00658.

[91] E. C. Herrmann and L. S. Kucera, “Antiviral Substances in Plants of the Mint Family (Labiatae). III. Peppermint (Mentha piperita) and other Mint Plants,” Proc. Soc. Exp. Biol. Med., 1967, doi: 10.3181/00379727-124-31874.

[92] T. Nagai, H. Yamada, Y. Suzuki, and T. Tomimori, “Antiviral Activity of Plant Flavonoid, 5,7,4′-Trihydroxy-8-Methoxyflavone, from the Roots of Scutellaria Baicalensis Against Influenza a (H3N2) and B Viruses,” Biol. Pharm. Bull., 1995, doi: 10.1248/bpb.18.295.

[93] R. M. Romeilah, S. A. Fayed, and G. I. Mahmoud, “Antioxidant and antiviral activities of essential oils from Callistemon viminalis and Schinus molle L,” Res. J. Pharm. Biol. Chem. Sci., 2016.

[94] M. D. R. Martins, S. Arantes, F. Candeias, M. T. Tinoco, and J. Cruz-Morais, “Antioxidant, antimicrobial and toxicological properties of Schinus molle L. essential oils,” J. Ethnopharmacol., 2014, doi: 10.1016/j.jep.2013.10.063.

[95] B. N. Dhawan, “Anti-viral activity of Indian plants,” Proceedings of the National Academy of Sciences India Section B - Biological Sciences. 2012, doi: 10.1007/s40011-011-0016-7.

[96] B. H. Tai et al., “An evaluation of the RNase H inhibitory effects of Vietnamese medicinal plant extracts and natural compounds,” Pharm. Biol., 2011, doi: 10.3109/13880209.2011.563316.

[97] C. P. Khare, Indian Medicinal Plants, An Illustrated Dictionary.Berlin/Heidelberg. 2007.

[98] A. M. Allahverdiyev et al., “Development of New Antiherpetic Drugs Based on Plant Compounds,” in Fighting Multidrug Resistance with Herbal Extracts, Essential Oils and their Components, 2013.

[99] Y. X. Li, Y. B. Liu, A. Q. Ma, Y. Bao, M. Wang, and Z. L. Sun, “In vitro antiviral, anti-inflammatory, and antioxidant activities of the ethanol extract of Mentha piperita L.,” Food Sci. Biotechnol., 2017, doi: 10.1007/s10068-017-0217-9.

[100] S. Saleem et al., “Plants Fagonia cretica L. and Hedera nepalensis K. Koch contain natural compounds with potent dipeptidyl peptidase-4 (DPP-4) inhibitory activity,” J. Ethnopharmacol., 2014, doi: 10.1016/j.jep.2014.08.017.

[101] M. M. Pinheiro, A. Fabbri, and M. Infante, “Cytokine storm modulation in COVID-19: a proposed role for vitamin D and DPP-4 inhibitor combination therapy (VIDPP-4i),” Immunotherapy, 2021, doi: 10.2217/imt-2020-0349.

[102] M. Akram, S. Muhammad, A. Shah, and M. Daniyal, “Antiviral potential of medicinal plants against HIV , HSV , influenza , hepatitis , and coxsackievirus : A systematic review,” no. February 2017, pp. 1–12, 2018, doi: 10.1002/ptr.6024.

[103] P. Indrasetiawan et al., “Antiviral activity of cananga odorata against hepatitis B virus,” Kobe J. Med. Sci., vol. 65, no. 2, pp. E71–E79, 2019.

[104] K. A. El Sayed*, “Natural Products as Antiviral Agents,” 2007, doi: https://doi.org/10.1016/S1572-5995(00)80051-4.

[105] R. V. Chikhale et al., “In-silico investigation of phytochemicals from Asparagus racemosus as plausible antiviral agent in COVID-19,” J. Biomol. Struct. Dyn., vol. 0, no. 0, pp. 1–15, 2020, doi: 10.1080/07391102.2020.1784289.

[106] K. Dhama et al., "Medicinal and Therapeutic Potential of Herbs and Plant Metabolites / Extracts Countering Viral Pathogens – Current Knowledge and Future Prospects," Curr. Drug Metab., vol. 19, pp. 236–263, 2018, doi: 10.2174/1389200219666180129145252.

[107] M. Mukhtar, M. Arshad, M. Ahmad, R. J. Pomerantz, B. Wigdahl, and Z. Parveen, "Antiviral potentials of medicinal plants," Virus Res., vol. 131, no. 2, pp. 111–120, 2008, doi: 10.1016/j.virusres.2007.09.008.

[108] G. Kumar Anbazhagan, S. Palaniyandi, and B. Joseph, "Antiviral Plant Extracts," Plant Extr., pp. 1–10, 2019, doi: 10.5772/intechopen.85126.

[109] S. K. Lam and T. B. Ng, "A protein with antiproliferative, antifungal and HIV-1 reverse transcriptase inhibitory activities from caper (Capparis spinosa) seeds," Phytomedicine, vol. 16, no. 5, pp. 444–450, 2009, doi: 10.1016/j.phymed.2008.09.006.

[110] H. Zhang and Z. F. Ma, "Phytochemical and pharmacological properties of capparis spinosa as a medicinal plant," Nutrients, vol. 10, no. 2, pp. 1–14, 2018, doi: 10.3390/nu10020116.

[111] O. Callies et al., "Isolation, structural modification, and HIV inhibition of pentacyclic lupane-type triterpenoids from cassine xylocarpa and maytenus cuzcoina," J. Nat. Prod., vol. 78, no. 5, pp. 1045–1055, 2015, doi: 10.1021/np501025r.

[112] D. Mathew and W. L. Hsu, "Antiviral potential of curcumin," J. Funct. Foods, vol. 40, no. September 2017, pp. 692–699, 2018, doi: 10.1016/j.jff.2017.12.017.

[113] M.-T. Moradi, M. Rafieian-Kopaei, and A. Karimi, "A review study on the effect of Iranian herbal medicines against in vitro replication of herpes simplex virus.," Avicenna J. phytomedicine, vol. 6, no. 5, pp. 506–515, 2016, doi: 10.22038/ajp.2016.6567.

[114] K. Ueda, R. Kawabata, T. Irie, Y. Nakai, Y. Tohya, and T. Sakaguchi, "Inactivation of Pathogenic Viruses by Plant-Derived Tannins: Strong Effects of Extracts from Persimmon (Diospyros kaki) on a Broad Range of Viruses," PLoS One, vol. 8, no. 1, pp. 1–10, 2013, doi: 10.1371/journal.pone.0055343.

[115] S. Ben-Shabat, L. Yarmolinsky, D. Porat, and A. Dahan, "Antiviral effect of phytochemicals from medicinal plants: Applications and drug delivery strategies," Drug Deliv. Transl. Res., vol. 10, no. 2, pp. 354–367, 2020, doi: 10.1007/s13346-019-00691-6.

[116] R. Iberahim, N. S. M. Nor, W. A. Yaacob, and N. Ibrahim, "Eleusine indica inhibits early and late phases of herpes simplex virus type 1 replication cycle and reduces progeny infectivity," Sains Malaysiana, vol. 47, no. 7, pp. 1431–1438, 2018, doi: 10.17576/jsm-2018-4707-10.

[117] L. Yarmolinsky, M. Huleihel, M. Zaccai, and S. Ben-Shabat, "Potent antiviral flavone glycosides from Ficus benjamina leaves," Fitoterapia, vol. 83, no. 2, pp. 362–367, 2012, doi: 10.1016/j.fitote.2011.11.014.

[118] L. Yarmolinsky, "Anti-viral activities of Ficus benjamina and Lilium candidum . Beer-Sheva Anti-viral activities of Ficus benjamina and Lilium candidum . Approved by the advisors : Prof . Mahmoud Huleihel ________________ Approved by the Dean of the Kreitman School of Advanced Graduate Studies," pp. 1–86, 2012.

[119] L. Cahlíková, K. Breiterová, and L. Opletal, "Chemistry and biological activity of alkaloids from the genus lycoris (Amaryllidaceae)," Molecules, vol. 25, no. 20, 2020, doi: 10.3390/molecules25204797.

[120] M. T. Islam et al., "Natural products and their derivatives against coronavirus: A review of the non-clinical and pre-clinical data," Phyther. Res., vol. 34, no. 10, pp. 2471–2492, 2020, doi: 10.1002/ptr.6700.

[121] C. Y. Fang et al., "Honokiol, a lignan biphenol derived from the Magnolia tree, inhibits dengue virus type 2 infection," Viruses, vol. 7, no. 9, pp. 4894–4910, 2015, doi: 10.3390/v7092852.

[122] H. Luo et al., "A review of the phytochemistry and pharmacological activities of Magnoliae officinalis cortex," J. Ethnopharmacol., vol. 236, no. February, pp. 412–442, 2019, doi: 10.1016/j.jep.2019.02.041.

[123] J. Malayan, B. Selvaraj, A. Warrier, S. Shanmugam, M. Mathayan, and T. Menon, "Anti-mumps virus activity by extracts of Mimosa pudica, a unique Indian medicinal plant," Indian J. Virol., vol. 24, no. 2, pp. 166–173, 2013, doi: 10.1007/s13337-013-0143-2.

[124] M. J. Ruffa et al., "Antiviral activity of Petiveria alliacea against the bovine viral diarrhea virus," Chemotherapy, vol. 48, no. 3, pp. 144–147, 2002, doi: 10.1159/000064920.

[125] M. Gupta and J. Vaghela, "Recent Advances in Pharmacological and Phytochemistry Studies on Phyllanthus amarus," Pharm. Biosci. J., vol. 7, no. 1, p. 01, 2019, doi: 10.20510/ukjpb/7/i1/179295.

[126] E. M. D. S. Siqueira et al., "Antiviral Potential of Spondias mombin LLeaves Extract against Herpes Simplex Virus Type-1 Replication Using in Vitro and in Silico Approaches," Planta Med., vol. 86, no. 7, pp. 505–515, 2020, doi: 10.1055/a-1135-9066.

[127] S. Sameh, E. Al-Sayed, R. M. Labib, and A. N. Singab, " Genus Spondias : A Phytochemical and Pharmacological Review ," Evidence-Based Complement. Altern. Med., vol. 2018, pp. 1–13, 2018, doi: 10.1155/2018/5382904.

[128] L. M. Bedoya, S. Sanchez-Palomino, M. J. Abad, P. Bermejo, and J. Alcami, "Anti-HIV activity of medicinal plant extracts," J. Ethnopharmacol., vol. 77, no. 1, pp. 113–116, 2001, doi: 10.1016/S0378-8741(01)00265-3.
